# Hypervesiculation Meets Sec-Targeting: Enhancing Heterologous Protein Loading in *Salmonella* Typhi Outer Membrane Vesicles for Delivery and Immune Response

**DOI:** 10.3390/ijms26094223

**Published:** 2025-04-29

**Authors:** Ignacio Fuentes, Francisco Parra, Diego Rojas, Andrés Silva, Jan Nevermann, María Carolina Otero, Fernando Gil, Iván L. Calderón, Juan A. Fuentes

**Affiliations:** 1 Laboratorio de Genética y Patogénesis Bacteriana, Centro de Investigación de Resiliencia a Pandemias, Facultad de Ciencias de la Vida, Universidad Andres Bello, Santiago 8370186, Chile; i.fuentescceres@uandresbello.edu (I.F.); f.parralathrop@uandresbello.edu (F.P.); d.rojasjacob@uandresbello.edu (D.R.); a.silvapeailillo@uandresbello.edu (A.S.); j.nevermann@uandresbello.edu (J.N.); 2 Doctorado en Biotecnología, Facultad de Ciencias de la Vida, Universidad Andres Bello, Santiago 8370186, Chile; 3 Escuela de Química y Farmacia, Facultad de Medicina, Universidad Andres Bello, Santiago 7591538, Chile; maria.otero@unab.cl; 4 School of Medicine, Faculty of Medicine, Universidad de los Andes, Santiago 7620001, Chile; frgil@uandes.cl; 5 Microbiota-Host Interactions & Clostridia Research Group, Center for Biomedical Research and Innovation (CIIB), Universidad de los Andes, Santiago 7620001, Chile; 6 Laboratorio de RNAs Bacterianos, Centro de Investigación de Resiliencia a Pandemias, Facultad de Ciencias de la Vida, Universidad Andres Bello, Santiago 8370186, Chile

**Keywords:** OMVs, *Salmonella* Typhi, hypervesiculation, Sec-targeting, heterologous protein loading, mCherry, *tolR*, *degS*, nanocarriers, immunogenicity

## Abstract

*Salmonella enterica* serovar Typhi (*S.* Typhi) produces outer membrane vesicles (OMVs) that remain comparatively underexplored as potential biotechnological tools. Here, we investigated how hypervesiculating *S.* Typhi mutants (Δ*tolR* and Δ*degS*) can be engineered to load and deliver the fluorescent reporter protein mCherry, targeting human epithelial cells and the murine immune system. Deletions in *tolR* and *degS* led to distinct OMV phenotypes characterized by higher vesicle production and altered cargo composition, underscoring the impact of disrupted membrane integrity and envelope stress on OMV biogenesis. By fusing mCherry with the *S.* Typhi OmpA signal peptide (SP*_ompA_*), we achieved robust and functionally intact intravesicular packaging in all strains. Flow cytometry and confocal microscopy revealed that the Δ*tolR* mutant exhibited particularly high cargo loading in the OMV fraction and pronounced mCherry delivery to epithelial cells, highlighting the potential of hypervesiculation to enhance OMV-based protein transport. However, immunization studies in mice showed that wild-type OMVs, despite carrying less mCherry than their hypervesiculating counterparts, induced the strongest anti-mCherry IgG responses. These findings indicate that, at least under these conditions, antigen loading alone is not sufficient to fully determine immunogenicity. Instead, the intrinsic composition or adjuvant-like properties of OMVs play a pivotal role in driving robust immune activation. Our results establish *S.* Typhi OMVs, especially when genetically modified with a Sec-dependent targeting signal (SP*_ompA_*), as versatile platforms for heterologous protein delivery. Although hypervesiculation facilitates increased protein encapsulation and delivery to epithelial cells, native OMVs appear to better preserve and/or present antigens for effective immunogenic responses in vivo. These insights set the stage for further optimization of *S.* Typhi OMVs in vaccine development and protein therapeutics, where balancing cargo loading with immunostimulatory features may be key to achieving maximal efficacy.

## 1. Introduction

Bacterial outer membrane vesicles (OMVs) are nanoscale, lipid-based spherical structures (20–250 nm in diameter) secreted by Gram-negative bacteria [1,2]. These vesicles encapsulate various biomolecules, including lipopolysaccharides (LPSs), peptidoglycan fragments, proteins, lipids, nucleic acids, and small regulatory RNAs [3]. OMVs functionally contribute to nutrient acquisition, stress adaptation, virulence factor delivery, immune modulation, inflammatory responses, and antibiotic resistance [1,2,4,5,6]. Due to their nanoscale size and immunogenic components (e.g., LPSs and lipoproteins), OMVs have increasingly been explored as versatile biotechnological tools with applications in drug delivery and vaccine development, among others [7,8].

Their nanoscale dimensions facilitate efficient cellular uptake, while their intrinsic adjuvant properties can enhance immune activation [2,9,10]. Moreover, OMVs can be genetically or biochemically tailored to incorporate specific proteins or antigens, broadening their biotechnological utility. For example, OMVs have been engineered as vaccine platforms capable of inducing robust humoral and cellular immune responses without the pathogenic risks of live bacteria [2,9,10]. Despite their potential, current challenges related to production efficiency, scalability, and heterologous protein incorporation limit the broader use of OMVs in biotechnology [11].

Genetic engineering of OMV-producing strains represents a promising strategy to overcome these barriers [2,8]. By targeting the genes responsible for OMV biogenesis, one can boost production levels and also customize OMV cargo [2,8,11]. For instance, deleting genes such as *nlpI* and *mlaE* in *Escherichia coli* can yield hypervesiculating mutants that produce up to 30-fold more OMVs than wild-type strains [12]. However, these mutations often disrupt outer membrane stability, stress response pathways, or vesiculation processes, leading to hypervesiculation [12,13,14]. While hypervesiculation substantially increases OMV output, it also alters OMV properties; in comparison to wild-type bacteria, mutant-derived OMVs frequently exhibit differences in size, composition, and functionality [13,15,16,17]. Consequently, it is critical to fully characterize these modified OMVs to understand their scope and limitations.

In addition, OMV content can be further manipulated by engineering strains to incorporate heterologous proteins, thereby expanding OMV functionality [11,18,19]. Selecting an appropriate signal peptide (SP) is essential for ensuring proper protein targeting and stability, as not all SPs efficiently guide heterologous proteins into OMVs [18,19,20,21]. Studies in *E. coli* have shown that while certain SPs (e.g., factor H-binding protein, fHbp from *Neisseria meningitidis*) successfully direct proteins, such as GFPmut2, to OMVs, others prove ineffective [21]. Thus, achieving effective protein incorporation is challenging and requires a detailed understanding of bacterial secretion and OMV biogenesis [13,15,16,22]. Further research is warranted to refine OMV engineering strategies and maximize yields of functional OMVs for biotechnological use.

OMVs from *Salmonella enterica* subspecies *enterica* serovar Typhi (*S.* Typhi), the etiological agent of typhoid fever, offer a promising yet underexplored system for such applications [13,15,16,22]. *S.* Typhi is a Gram-negative bacterium that causes systemic infection following the ingestion of contaminated food or water [23,24]. Once internalized, *S.* Typhi crosses the intestinal epithelium and disperses throughout the body, targeting organs such as the liver, spleen, and bone marrow [25,26,27]. Although vaccines and antibiotics are available, typhoid fever still poses a significant global burden, with an estimated 9.2 million cases and 110,000 deaths in 2019, predominantly in Southeast Asia, the Eastern Mediterranean, and Africa [27,28,29,30,31]. The surge in multidrug-resistant *S.* Typhi further emphasizes the need for novel therapeutic and preventive strategies [32].

The OMVs of *S.* Typhi are particularly compelling due to the unique adaptive strategies of this human-restricted pathogen, distinguishing it from well-studied serovars such as *Salmonella* Typhimurium [33,34,35]. During reductionist evolution, *S.* Typhi lost several proinflammatory factors, such as SopA, facilitating immune evasion and reduced intestinal inflammation [31,36]. In addition, *S.* Typhi expresses the Vi capsular antigen via the SPI-7 pathogenicity island, which helps suppress Toll-like receptor (TLR) activation, mitigate early immune responses, and promote chronic infections [31,37,38]. Such factors may affect the composition and immunomodulatory properties of *S.* Typhi OMVs; indeed, the Vi antigen itself is carried by these vesicles, potentially contributing distinctive immune-modifying capabilities [31,38].

Despite these intriguing biological features, *S.* Typhi OMVs remain understudied, particularly regarding their interactions with eukaryotic cells and suitability for heterologous protein delivery. Although they have been proposed as a vaccine platform against typhoid fever [31,39], their properties and applications remain poorly characterized. In particular, little is known about how these OMVs interact with eukaryotic cells or deliver heterologous proteins, and it remains unexamined whether they can elicit immune responses against these foreign antigens. To address these knowledge gaps, we evaluated OMVs derived from hypervesiculating *S.* Typhi mutants (∆*tolR* and ∆*degS*) [40] for their capacity to incorporate and deliver heterologous proteins. *tolR* encodes part of the Tol-Pal system that upholds outer membrane integrity [24,41], while *degS* encodes a serine protease that activates the σ^E^ envelope stress response [13,15]. Both mutants not only increase OMV production but also exhibit notable changes in OMV composition, size, and function compared to the wild type [13,15].

Here, we used the *ompA* (porin) signal peptide (SP*_ompA_*) to direct a model cytoplasmic protein, mCherry, into the lumen of OMVs produced by *S.* Typhi WT, ∆*tolR*, and ∆*degS*. We then examined whether these OMVs could deliver functional mCherry to HT-29 epithelial cells and induce anti-mCherry antibody responses in mice. Our results show that SP*_ompA_* enables efficient, functional loading of mCherry into OMVs from all tested strains. Notably, ∆*tolR*-derived OMVs displayed higher levels of mCherry loading in the OMV fraction and facilitated superior delivery to epithelial cells, although WT OMVs carrying mCherry elicited the most potent immune response in mice. Collectively, these findings establish the feasibility of using *S.* Typhi OMVs to deliver heterologous proteins and illustrate their potential as robust platforms for biotechnological applications.

## 2. Results

### 2.1. Characterization of OMVs Derived from Salmonella Typhi WT, ∆tolR, and ∆degS Mutants

The functional and structural properties of OMVs are inherently linked to their biogenesis, composition, and morphology [13,15]. While the OMVs derived from *S.* Typhi WT, ∆*tolR*, and ∆*degS* strains have previously been demonstrated to exhibit hypervesiculatory behavior [13], re-characterization in this study was essential to account for the unique experimental conditions and advanced analytical techniques applied here. This re-characterization was conducted to ensure the reproducibility and reliability of the observed OMV phenotypes under the specific parameters of our study. By employing an integrative approach that included detailed morphology assessment via TEM, size distribution analysis, and protein profiling through SDS-PAGE, we sought to provide a comprehensive and nuanced understanding of how genetic modifications in the *tolR* and *degS* genes affect OMV biogenesis and properties. These refined analyses offer enhanced resolution and sensitivity, enabling the identification of subtle but significant phenotypic distinctions that may have been overlooked in earlier studies.

**Figure 1:**
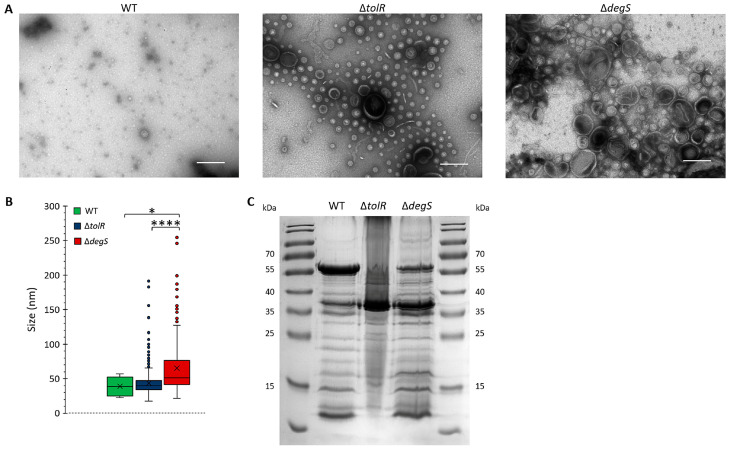
Characterization of OMVs derived from *Salmonella* Typhi WT, ∆*tolR*, and ∆*degS* strains. (**A**) Transmission electron microscopy (TEM) images of OMVs isolated from *S.* Typhi WT, ∆*tolR*, and ∆*degS* strains. Scale bars: 200 nm. The horizontal field width was set to 1.5 µm, and the magnification was 60,000×. A representative experiment is presented for each condition. (**B**) Size distribution of OMVs measured from TEM images. Box-and-whisker plots display the median size, interquartile range, and outliers. Measurements were obtained from three independent fields per strain for each biological replicate (n ≥ 3). Statistical analysis was performed using the Kruskal–Wallis test followed by Dunn′s post hoc test (* *p* < 0.05; **** *p* < 0.0001). One-way ANOVA followed by Tukey′s post hoc test was used to assess statistical significance (* *p* < 0.05; **** *p* < 0.0001). (**C**) SDS-PAGE analysis of OMV protein profile. Thirty micrograms of OMVs (quantified by their protein content) were resolved and stained.

Transmission electron microscopy (TEM) was used to assess the impact of *tolR* and *degS* deletions on OMV morphology (Figure 1A). The OMVs produced by the *S.* Typhi WT strain exhibited a sparse distribution, with vesicles displaying uniform, spherical shapes, and a consistent size range, indicative of tightly regulated vesiculation that maintains outer membrane stability under physiological conditions. In contrast, the ∆*tolR* mutant showed a significant increase in OMV density, with a broader size distribution, while maintaining a generally spherical morphology. The observed hypervesiculation aligns with the role of the Tol-Pal system in preserving outer membrane integrity; its disruption promotes compensatory vesicle release in response to membrane instability [13,15,17,41,42]. The ∆*degS* mutant exhibited the most pronounced vesiculation phenotype, characterized by a substantial increase in vesicle production and marked size heterogeneity. These vesicles were significantly larger and displayed greater structural variability than those derived from the WT and ∆*tolR* strains. The absence of DegS, a key regulator of the σ^E^ stress response, likely intensifies envelope stress, leading to excessive and dysregulated OMV production [13,15,43].

To quantify size differences, vesicle diameters from the TEM images were measured (Figure 1B). The OMVs from the *S.* Typhi WT strain exhibited the smallest and most uniform size distribution, with a median diameter of ~40 nm and a narrow interquartile range (IQR). The OMVs from the *S.* Typhi ∆*tolR* mutant were slightly larger, with a median diameter of ~45 nm and increased size variability, as evidenced by a broader IQR and outliers extending to ~200 nm. These features suggest greater size heterogeneity due to compromised outer membrane stability caused by the absence of TolR [41]. The *S.* Typhi ∆*degS* mutant produced the largest vesicles, with a median diameter of ~65 nm and the widest size variability. Numerous outliers exceeded 200 nm, further emphasizing the structural heterogeneity. This heightened variability likely reflects the extreme envelope stress caused by the loss of DegS, resulting in increased OMV biogenesis [43].

SDS-PAGE analysis of the OMV protein profiles revealed distinct changes associated with the *S.* Typhi ∆*tolR* and ∆*degS* mutations (Figure 1C). The deletion of *tolR* and *degS* influenced the incorporation of proteins into OMVs, reflecting alterations in vesicle biogenesis and outer membrane remodeling. This analysis provided insights into how the deletion of *tolR* and *degS* influences the incorporation of proteins into OMVs, reflecting alterations in vesicle biogenesis and outer membrane remodeling.

The results demonstrate that genetic disruptions in the Tol-Pal system (*tolR*) and the σ^E^ stress response (*degS*) significantly influence OMV biogenesis, leading to altered morphology, increased size heterogeneity, enhanced production, and modified protein composition. These findings align with previous studies highlighting the roles of TolR and DegS in OMV biogenesis and underscore their distinct contributions to vesicle structure and function. The enhanced yield and altered properties of the OMVs derived from the mutant strains suggest their potential utility for biotechnological applications, such as heterologous protein delivery and vaccine development.

### 2.2. Efficient Targeting of mCherry to OMVs Using the S. Typhi ompA Signal Peptide (SP_ompA_)

OmpA, a conserved outer membrane protein, is consistently identified in OMVs from various bacteria, including enterobacteria such as *Escherichia coli* [44,45]. In *E. coli*, OmpA has been successfully used to direct epitopes to OMVs by fusing a FLAG to its β-barrel domain, specifically at the C-terminal end, up to the 174th residue located on the periplasmic side [46]. In this study, we sought to evaluate the potential of the *S.* Typhi OmpA signal peptide (SP*_ompA_*), a short sequence of only 21 residues (MKKTAIAIAVALAGFATVAQA), as a targeting signal for directing proteins to OMVs. Signal peptides are known to enhance the secretion efficiency of fusion proteins, either for periplasmic localization or extracellular export, through careful design and optimization [47,48]. To the best of our knowledge, the use of the *S.* Typhi OmpA signal peptide for targeting fusion proteins to OMVs has not been previously reported, particularly in the context of mutant genetic backgrounds that alter OMV biogenesis, such as Δ*tolR* and Δ*degS*.

To test the efficiency of SP*_ompA_* in targeting fusion proteins into OMVs, we employed mCherry as a reporter protein. In this context, a key advantage of mCherry is its ability to fold into an active conformation in the periplasm, unlike many GFP derivatives and other fluorescent proteins [49]. Upon fusion with SP*_ompA_*, mCherry is expected to be recruited as an unfolded, chaperone-stabilized (e.g., via SecB) protein to the Sec translocase, which directs its translocation to the periplasm. There, under oxidizing conditions, mCherry can properly fold and maintain its fluorescence [50,51], enabling reliable monitoring of protein targeting and potential functionality in OMVs.

To investigate the incorporation of mCherry into *S.* Typhi OMVs, two constructs were generated, i.e., a cytoplasmic mCherry (*mcherry*) and another encoding an SP*_ompA_*-*mcherry* fusion, in which the *S.* Typhi OmpA signal peptide (SP*_ompA_*) was appended to the N-terminus of mCherry. Both constructs were cloned into the pBAD33-GM vector under the control of the P*_araBAD_* promoter, enabling arabinose-inducible expression and resulting in the pB*mcherry* and pBSP*_ompA_*-*mcherry* plasmids, respectively (Figures S1 and S2). A C-terminal FLAG tag was added to mCherry in both constructs to facilitate detection. Correct plasmid assembly was confirmed by restriction digestion and sequencing.

The incorporation of mCherry into OMVs was evaluated using Western blot analysis. OMVs were isolated from the *S.* Typhi WT, ∆*tolR*, and ∆*degS* strains transformed with plasmids expressing either cytoplasmic mCherry (pB*mcherry*) or mCherry fused to the *S.* Typhi OmpA signal peptide (SP*_ompA_*) (pBSP*_ompA_*-*mcherry*). The protein content was standardized across all samples (30 µg per lane). The Western blot for mCherry revealed clear differences in the levels of incorporation depending on the strain and construct.

**Figure 2:**
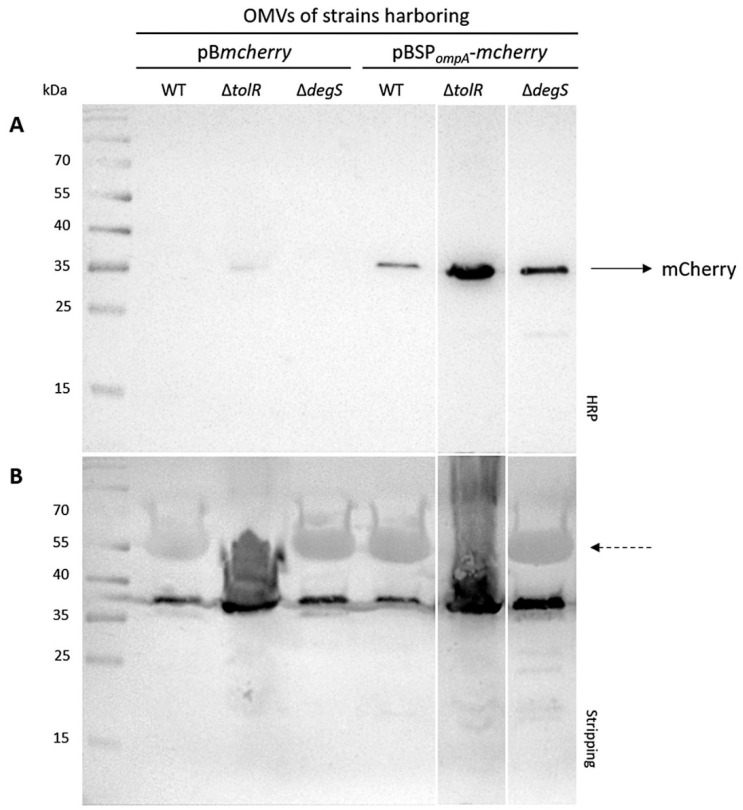
Incorporation of mCherry into OMVs from *S.* Typhi WT, ∆*tolR*, and ∆*degS* strains. (**A**) Western blot analysis of OMVs isolated from *S.* Typhi WT, ∆*tolR*, and ∆*degS* strains transformed with plasmids encoding cytoplasmic mCherry (pB*mcherry*) or mCherry fused to the *S.* Typhi OmpA signal peptide (SP*_ompA_*, pBSP*_ompA_*-*mcherry*). OMVs were isolated from arabinose-induced cultures, and protein content was standardized to 30 µg per lane. Detection was performed using an anti-FLAG antibody targeting the C-terminal FLAG tag in mCherry. (**B**) The membrane was stripped and re-probed with a polyclonal antibody raised against *S.* Typhi WT OMVs to confirm equal OMV protein loading across all samples. The uniform signal intensity observed across all lanes validates the comparability of mCherry incorporation levels and confirms consistent OMV yields between strains and constructs. Notably, a protein of approximately 55 kDa was not detected in OMVs from the ∆*tolR* mutant, according to the results shown in Figure 1C. This protein is presumably flagellin, whose absence correlates with the low motility (swimming) observed in the ∆*tolR* strain (Figure S3). The uniform signal intensity of other OMV-associated proteins across all lanes confirms comparable OMV yields between strains and constructs. The arrow with the dashed line could potentially correspond to flagellin. The Western blot shown is representative of two independent experiments (n = 3).

No mCherry band was detected in the OMVs isolated from the *S.* Typhi WT and ∆*degS* strains harboring the pB*mcherry* plasmid. However, a faint mCherry band was observed in the OMVs of the ∆*tolR* mutant carrying pB*mcherry* (Figure 2A). In contrast, a clear and consistent mCherry band was observed in the OMVs derived from all *S.* Typhi strains (WT, ∆*tolR*, and ∆*degS*) harboring the pBSP*_ompA_*-*mcherry* plasmid (Figure 2A). This strongly supports the functionality of the SP*_ompA_* signal peptide in directing mCherry to OMVs. SP*_ompA_* likely facilitates the translocation of mCherry into the periplasmic space, from which it is efficiently incorporated into vesicles. Notably, the presence of a robust mCherry band across all strains indicates that this targeting mechanism operates consistently, regardless of the genetic background. These findings confirm that cytoplasmic mCherry alone is not efficiently incorporated into OMVs without a targeting mechanism, highlighting the necessity of the SP*_ompA_* signal peptide for successful incorporation. To ensure accurate comparisons, the Western blot membrane was stripped and re-probed with a polyclonal antibody raised against the OMVs from the *S.* Typhi WT strain, as a loading control. Uniform signal intensity was observed across all lanes, confirming that similar amounts of OMV protein were loaded for each sample (Figure 2B). This consistency validates the comparability of mCherry incorporation levels. Overall, these results demonstrate that the SP*_ompA_* signal peptide significantly enhances the incorporation of mCherry into OMVs. This effect could potentially be further amplified in hypervesiculating mutant strains, which may yield higher OMV production and enhanced incorporation efficiency.

It is important to note that loading 30 µg of total OMV protein does not necessarily correspond to the same number of vesicles across samples, particularly given the differences in OMV size and composition between the WT and mutants. This difference is likely due to the hypervesiculation phenotype in the ∆*tolR* and Δ*degS* strains, which produces a higher number of OMVs, consequently presenting a higher amount of mCherry in the OMV fraction. The Western blot analysis demonstrates that mCherry is successfully incorporated into OMVs when expressed with the *S.* Typhi OmpA signal peptide (SP*_ompA_*), as observed in the strains transformed with the plasmid pBSP*_ompA_*-*mcherry*. Notably, the incorporation of mCherry is more pronounced in the OMVs derived from the ∆*tolR* and ∆*degS* mutant strains compared to the WT strain. In contrast, the strains transformed with pB*mcherry*, encoding cytoplasmic mCherry lacking SP*_ompA_*, do not package mCherry into OMVs, except for the ∆*tolR* mutant, which shows a faint mCherry signal. These findings confirm that SP*_ompA_* is an effective targeting sequence for directing heterologous proteins, such as mCherry, into the OMVs across the tested *S.* Typhi strains.

### 2.3. SP_ompA_ Enhances the Functional Incorporation of mCherry into OMVs

To assess whether the fusion of mCherry with the *S.* Typhi OmpA signal peptide (SP*_ompA_*) affects its functionality, we evaluated the fluorescence of mCherry incorporated into the OMVs. Fluorescence is a key indicator of mCherry′s proper folding and functional state, which could potentially be influenced by the presence of SP*_ompA_*. To this end, the *S.* Typhi WT, ∆*tolR*, and ∆*degS* mutant strains were transformed with either the pB*mcherry* plasmid (encoding cytoplasmic mCherry without SP*_ompA_*) or the pBSP*_ompA_*-*mcherry* plasmid (encoding mCherry fused to SP*_ompA_*). Cultures of equal initial volumes were grown, and OMVs were extracted and concentrated by ultracentrifugation. The OMVs were concentrated 1000-fold relative to the original culture volume to ensure consistent handling and comparison. Fluorescence measurements were then performed on equivalent volumes of OMV suspensions from each condition. By standardizing the comparison based on culture volume, we aimed to evaluate OMV production efficiency and the incorporation of functional, fluorescent mCherry across the different strains and plasmid constructs. This approach allowed for a direct comparison of OMV fluorescence levels, providing insight into the impact of SP*_ompA_* on the functional incorporation of mCherry into OMVs.

**Figure 3:**
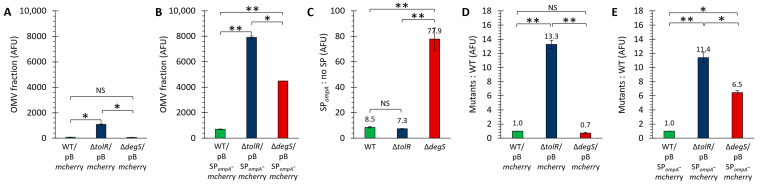
Fluorescence analysis of OMVs isolated from *S.* Typhi WT, ∆*tolR*, and ∆*degS* strains transformed with plasmids encoding cytoplasmic mCherry (pB*mcherry*) or mCherry fused to the *S.* Typhi OmpA signal peptide (SP*_ompA_*; pBSP*_ompA_*-*mcherry*). Fluorescence intensity was measured to assess the incorporation and functionality of mCherry within OMVs. Cultures of equal initial volumes were grown, OMVs were extracted, and fluorescence was evaluated (Exc: 587 nm; Em: 630 nm) from equivalent volumes of OMV suspensions. (**A**) Fluorescence of OMVs from strains carrying pB*mcherry*. (**B**) Fluorescence of OMVs from strains carrying pBSP*_ompA_*-*mcherry*. (**C**) Relative increase in fluorescence due to SP*_ompA_* in OMVs (pBSP*_ompA_*-*mcherry* vs. pB*mcherry*). Fluorescence of mutant strains compared to WT for pBmcherry (**D**) and pBSP*_ompA_*-*mcherry* (**E**). One-way ANOVA followed by Tukey′s post hoc test to determine statistical significance (n = 3, * *p* < 0.05, ** *p* < 0.001, NS: not significant).

Figure 3A shows the fluorescence intensity (arbitrary fluorescence units, AFUs) measured in the OMV fractions of the strains transformed with the plasmid pB*mcherry*. No significant fluorescence signal was detected for the WT strain, indicating that cytoplasmic mCherry is not efficiently incorporated into OMVs without a targeting mechanism. Similarly, the ∆*degS* mutant exhibited negligible fluorescence levels, suggesting that the absence of DegS does not lead to cytoplasmic mCherry leakage into OMVs. In contrast, the OMVs derived from the ∆*tolR* mutant displayed a significantly higher measurable fluorescence signal than the WT and ∆*degS* strains. These results align with the Western blot findings, where only the ∆*tolR* mutant showed a faint mCherry signal in the OMVs.

On the other hand, Figure 3B presents the fluorescence intensity (AFUs) measured in the OMV fractions of the strains transformed with the plasmid pBSP*_ompA_*-*mcherry*. The fluorescence levels in all three strains were significantly higher than their respective pB*mcherry* controls (Figure 3C), confirming that SP*_ompA_* effectively directs mCherry into OMVs. Among the strains, the OMVs from the ∆*tolR* mutant exhibited the highest fluorescence signal, followed by the ∆*degS* mutant and the WT strain. These results suggest that the hypervesiculating phenotypes of the ∆*tolR* and ∆*degS* mutants enhance the incorporation or retention of SP*_ompA_*-*mcherry* in the OMV fraction compared to the WT strain. This pattern is consistent with the Western blot data, where the OMVs from all strains harboring the pBSP*_ompA_*-*mcherry* plasmid showed robust mCherry signals, with the highest intensity in the ∆*tolR* strain. The findings further validate that SP*_ompA_* efficiently facilitates the functional incorporation of mCherry into the OMVs across all the tested strains, with enhanced fluorescence in hypervesiculating mutants due to increased OMV production and/or changes in OMV composition.

Figure 3C highlights the efficiency of the SP*_ompA_* signal peptide in directing mCherry into OMVs. In the WT and ∆*tolR* strains, SP*_ompA_* increases mCherry incorporation approximately 8-fold compared to cytoplasmic mCherry. However, in the ∆*degS* mutant, the increase is markedly higher (around 78-fold), indicating a significantly enhanced effect of SP*_ompA_* in this strain.

Figure 3D shows the relative fluorescence of the OMVs from the ∆*tolR* and ∆*degS* mutants compared to the WT strain for cytoplasmic mCherry (pB*mcherry*). The ∆*tolR* mutant displays a 13.3-fold increase in fluorescence, showing the presence of significant cytoplasmic proteins in the OMV fraction, while the ∆*degS* mutant shows no increase, with fluorescence comparable to the WT strain.

Figure 3E compares the fluorescence of SP*_ompA_*-*mcherry* in the OMVs from the mutant strains relative to the WT strain. The ∆*tolR* mutant shows an 11.4-fold increase, while the ∆*degS* mutant shows a 6.5-fold increase, both significantly higher than the WT strain. This indicates that SP*_ompA_* is more effective in facilitating mCherry incorporation into OMVs in hypervesiculating mutants, with ∆*tolR* showing the most significant enhancement.

These findings are qualitatively consistent with the Western blot results, where robust mCherry signals were observed in the OMVs from all the strains harboring SP*_ompA_*-*mcherry*, with the highest intensity in the hypervesiculating mutants, particularly ∆*tolR*. Furthermore, the absence of mCherry incorporation into the OMVs from the strains transformed with the cytoplasmic mCherry construct (pB*mcherry*), except for the low levels observed in ∆*tolR*, further reinforces the conclusion that SP*_ompA_* is crucial for the efficient targeting and packaging of mCherry into OMVs.

These results confirm that the fluorescence observed in OMVs is specific to functionally folded mCherry and that the SP*_ompA_* signal peptide is critical for its efficient incorporation and retention within OMVs.

### 2.4. SP_ompA_ Directs mCherry to the Lumen of OMVs

To determine whether mCherry localizes within the lumen of OMVs, where it would be protected from external factors, we performed a proteinase K protection assay [52,53]. This assay distinguishes between proteins exposed on the OMV surface, which are susceptible to protease digestion, and luminal proteins, which remain protected unless the membrane is disrupted. The OMVs from the *S.* Typhi WT, ∆*tolR*, and ∆*degS* strains carrying the pBSP*_ompA_*-*mcherry* plasmid were treated with proteinase K, either in the presence or absence of SDS, and mCherry detection was assessed by Western blotting and fluorescence measurements. This approach allowed us to evaluate the spatial localization of mCherry within the OMVs and determine whether SP*_ompA_* facilitates its incorporation into the protected lumen.

**Figure 4:**
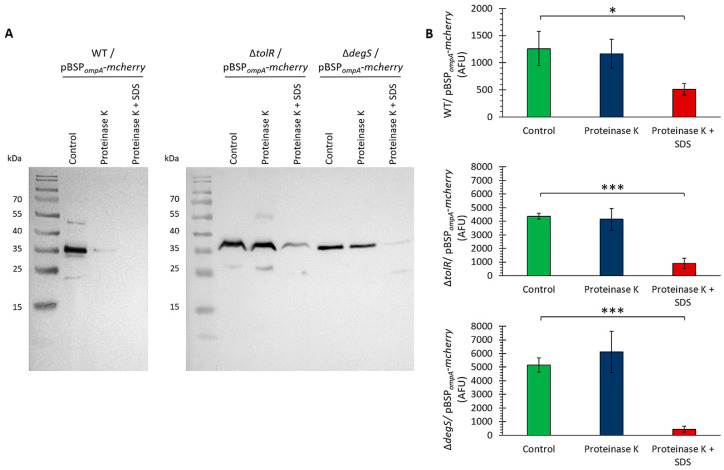
mCherry is localized within the lumen of OMVs. Purified OMVs from *S.* Typhi WT, ∆*tolR*, and ∆*degS* strains carrying the pBSP*_ompA_*-*mcherry* plasmid were incubated with proteinase K for 30 min at 37 °C, either in the absence or presence of 1% SDS to disrupt the OMV membrane. Untreated OMVs served as controls. After treatment, OMVs were analyzed by Western blotting (**A**) to detect mCherry or fluorescence measurements as an indicator of intact mCherry (**B**). The assays confirmed that mCherry is protected from protease digestion in intact OMVs and is degraded only upon membrane disruption with SDS, consistent with its localization in the lumen. Purified mCherry treated with SDS alone showed only about a 7.5% reduction in fluorescence (control, not shown in the graph) (n = 3; bars represent the standard error; one-way ANOVA with Dunnett’s post hoc test; * *p* < 0.05; *** *p* < 0.001).

Figure 4A shows that upon treatment with proteinase K alone, the mCherry band remained intact in the ∆*tolR* and ∆*degS* mutants, indicating that mCherry is fully protected within the OMV lumen in these strains. However, a slight reduction in the mCherry signal was observed in the WT strain, suggesting that a fraction of mCherry was accessible to protease digestion. When the OMVs were treated with proteinase K in the presence of SDS, which disrupts the OMV membrane, the mCherry band was almost completely degraded in all the strains. This shows that mCherry is localized within the lumen of OMVs, where it is protected from external protease activity unless the membrane integrity is compromised.

To further confirm the localization of mCherry within the lumen of the OMVs, a proteinase K protection assay was conducted using fluorescence as a readout instead of Western blotting. The OMVs were treated with proteinase K in the absence or presence of SDS, and fluorescence was measured as an indicator of intact mCherry. We obtained similar results (Figure 4B).

The combined results from the Western blot (Figure 4A) and fluorescence-based assay (Figure 4B) conclusively demonstrate that mCherry is protected from proteinase K degradation due to its localization in the lumen of OMVs. These findings confirm that the SP*_ompA_* signal peptide successfully targets mCherry to the lumen of OMVs, ensuring its protection from external environmental factors. This localization is crucial for potential applications requiring protein stability and functionality within OMVs. Together, these results underscore the potential of these OMVs as vehicles for the delivery of heterologous proteins.

### 2.5. Quantitative Analysis of mCherry-Loaded OMVs Across Strains

To determine the proportion of the OMVs carrying detectable levels of mCherry, we performed flow cytometry using the small particle side scatter (SP SSC-A) to analyze the OMVs directly based on their complexity and PE-Texas Red fluorescence to detect mCherry. This approach provides a quantitative assessment of mCherry incorporation into OMVs, allowing us to evaluate the efficiency of mCherry targeting mediated by SP*_ompA_* and compare the OMV populations derived from the *S.* Typhi WT, ∆*tolR*, and ∆*degS* strains. By combining complexity and fluorescence measurements, this experiment offers precise insight into the extent to which mCherry is packaged within OMVs under different genetic backgrounds and constructs.

**Figure 5:**
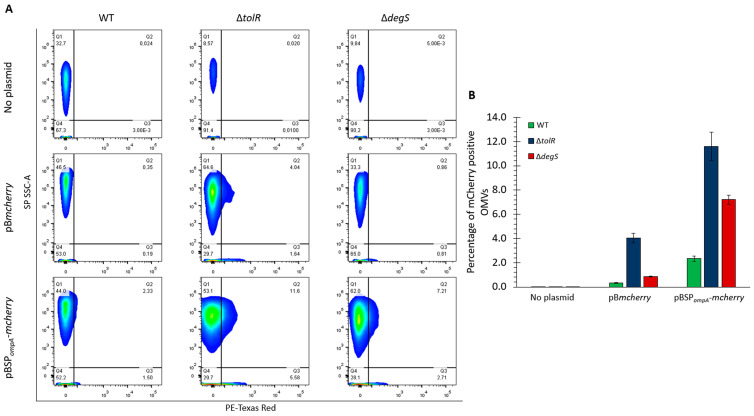
Quantitative analysis of mCherry-loaded OMVs using flow cytometry. OMVs purified from *S.* Typhi WT, ∆*tolR*, and ∆*degS* strains under different experimental conditions (no plasmid, pB*mcherry*, or pBSP*_ompA_*-*mcherry*) were analyzed by flow cytometry. Small particle side scatter (SP SSC-A) was used to identify OMVs based on their complexity, while PE-Texas Red fluorescence was used to detect mCherry within OMVs. (**A**) Representative dot plots showing the complexity (SP SSC-A) and fluorescence (PE-Texas Red) profiles for OMVs from each strain and condition. This was a representative experiment (n = 3). (**B**) Quantification of the percentage of mCherry-positive OMVs across strains and constructs. This approach enabled the evaluation of SP*_ompA_*-mediated mCherry incorporation into OMVs and the comparison of mCherry packaging efficiency across genetic backgrounds. The bars correspond to the standard error (n = 3).

Figure 5A shows that in the absence of a plasmid (no plasmid control), no significant fluorescence was detected in the PE-Texas Red channel across any strain, confirming the lack of mCherry in these OMVs. For the OMVs derived from the strains carrying the pB*mcherry* plasmid, low fluorescence was observed in the ∆*tolR* mutant, with 4.04% of the OMVs showing detectable mCherry levels, consistent with our previous results. In contrast, the OMVs from the WT and ∆*degS* strains with pB*mcherry* showed minimal fluorescence, indicating negligible mCherry incorporation without a targeting signal. In the OMVs derived from strains carrying the pBSP*_ompA_*-*mcherry* plasmid, a substantial increase in the proportion of fluorescent OMVs was observed. For the ∆*tolR* mutant, 11.6% of the OMVs were positive for mCherry, the highest percentage among the tested strains. The OMVs from the ∆*degS* mutant also showed a notable increase, with 7.21% of the OMVs carrying mCherry. The WT strain exhibited a lower percentage of fluorescent OMVs (2.33%) than the hypervesiculating mutants. Figure 5B shows the proportion of mCherry-positive OMVs from the *S.* Typhi WT, ∆*tolR*, and ∆*degS* strains under each condition (no plasmid, pB*mcherry*, or pBSP*_ompA_*-*mcherry*), as determined by flow cytometry for direct comparison.

These experiments are qualitatively consistent with the results of previous assays, including Western blotting (Figure 2) and fluorescence-based analysis of the OMVs (Figure 3). In all cases, the flow cytometry findings align with the observed trends in mCherry incorporation and functionality, further confirming that SP*_ompA_* effectively directs mCherry into OMVs and that hypervesiculating strains, particularly ∆*tolR*, exhibit the highest efficiency in packaging mCherry in the OMV fraction.

### 2.6. Optimizing mCherry Transport to Eukaryotic Cells Using OMVs from S. Typhi Hypervesiculatory Mutants

Understanding the interaction of OMVs with epithelial cells is essential to evaluate their potential as delivery systems for heterologous proteins, such as mCherry. While OMVs from *Escherichia coli* and *Salmonella* Typhimurium, among other bacterial species [54,55,56], have been extensively reported to internalize into epithelial cells, thereby facilitating processes such as host-pathogen interactions and immune modulation, the internalization of OMVs derived from *Salmonella* Typhi remains unreported in the literature. This knowledge gap is particularly relevant due to the unique adaptations of *S.* Typhi, including its human-restricted host range and immunomodulatory capabilities [31,36]. To address this, we employed HT-29 cells, a well-established epithelial cell model [54], to investigate whether the OMVs from *S.* Typhi, including those produced by the ∆*tolR* and ∆*degS* hypervesiculatory mutants, can internalize into epithelial cells and potentially serve as carriers for heterologous proteins.

To track OMV uptake, the OMVs derived from the *S.* Typhi WT, ∆*tolR*, and ∆*degS* strains were labeled with the lipophilic dye DiO (green), while cell nuclei were counterstained with Hoechst (blue) and cell membranes with WGA (red) to provide spatial localization. Confocal microscopy was used to visualize OMV-cell interactions following 30 min and 3 h of incubation. The analysis revealed that the DiO-labeled OMVs were successfully internalized by the HT-29 cells at both time points (Figure S4). At 30 min, OMVs were observed near the cell periphery, with partial co-localization with the cell membrane (red), indicative of early interactions or initial stages of endocytosis. After 3 h, the DiO signal was predominantly localized within the cytoplasm, confirming the successful internalization of the OMVs.

Assessing cell viability is critical to determine whether OMVs from *Salmonella* Typhi, including the ∆*tolR* and ∆*degS* mutants, induce cytotoxic effects on epithelial cells. Confirming that they preserve cellular integrity is essential, given their potential as delivery platforms for heterologous proteins. HT-29 cells were treated with OMVs from the *S.* Typhi WT, ∆*tolR*, and ∆*degS* strains (100 µg/mL) for 30 min and 3 h. As a control, the cells were incubated without OMVs to establish baseline viability. Cell viability was evaluated via propidium iodide (PI) staining, with PI-positive cells indicating compromised membrane integrity. Flow cytometry quantified the proportion of PI-positive cells. At 30 min, cell viability rates were 96.8% in the control and 91.0%, 92.5%, and 94.1% in the WT, ∆*tolR*, and ∆*degS* OMVs, respectively, showing minimal cytotoxicity. After 3 h, the viability slightly declined to 90.9% in the control and 92.6%, 92.6%, and 93.9% in the WT, ∆*tolR*, and ∆*degS* OMVs, but it remained consistently above 90% across all groups (Figure S5). These findings demonstrate that OMVs from *S.* Typhi WT and hypervesiculatory mutants have negligible cytotoxicity on HT-29 cells, comparable to untreated controls, supporting their potential as delivery platforms for biotechnological applications.

As previously established, OMVs from *S.* Typhi effectively internalize into HT-29 epithelial cells and exhibit negligible cytotoxic effects under the tested conditions. Building on this foundation, we evaluated the ability of OMVs from *S.* Typhi to deliver heterologous proteins into eukaryotic cells. Confocal microscopy was used to visualize the internalization of mCherry-loaded OMVs in HT-29 epithelial cells. The OMVs derived from the *S.* Typhi WT, ∆*tolR*, and ∆*degS* mutants harboring pB*mcherry* or pBSP*_ompA_*-*mcherry* constructs were fluorescently labeled with DiO (green) and incubated with HT-29 cells. mCherry fluorescence (red) was used to track the delivery of the protein, while Hoechst staining (blue) marked cell nuclei.

**Figure 6:**
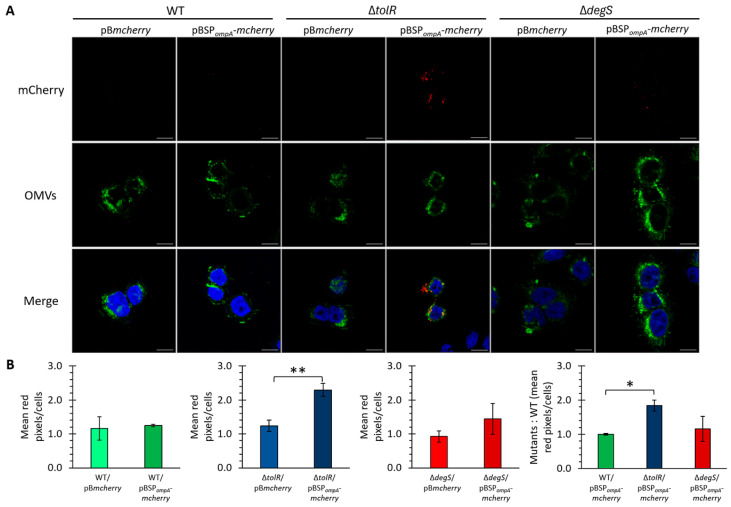
Delivery of mCherry-loaded OMVs into HT-29 epithelial cells. (**A**) Representative confocal microscopy images of HT-29 epithelial cells incubated with OMVs derived from *S.* Typhi WT, ∆*tolR*, and ∆*degS* strains carrying either the pB*mcherry* (encoding cytoplasmic mCherry) or pBSP*_ompA_*-*mcherry* (encoding mCherry fused to the SP*_ompA_*) constructs. OMVs are labeled with the lipophilic dye DiO (green), while mCherry fluorescence (red) indicates the protein cargo. Nuclei are stained with Hoechst (blue). Scale bars: 10 µm. (**B**) Quantification of mCherry fluorescence in HT-29 cells. The mean red pixel intensity per cell was calculated using Fiji software version 2.14 from at least 10 fields of view per biological replicate (n = 3). Data are presented as mean ± standard error. Statistical analysis was performed using one-way ANOVA with Tukey’s post hoc test (* *p* < 0.05, ** *p* < 0.01).

The confocal images revealed distinct differences in mCherry delivery efficiency between the strains and constructs (Figure 6A). For the OMVs derived from the strains carrying pB*mcherry*, no significant mCherry signal was observed within the HT-29 cells across all the strains (WT, ∆*tolR*, and ∆*degS*), consistent with the lack of a targeting mechanism for directing cytoplasmic mCherry into OMVs. In contrast, the OMVs derived from the strains carrying the pBSP*_ompA_*-*mcherry* construct displayed notable differences in mCherry delivery efficiency among the tested strains. No significant mCherry fluorescence was observed in the HT-29 cells treated with the OMVs from the WT strain, suggesting minimal protein delivery under these conditions. In stark contrast, the OMVs from the ∆*tolR* mutant demonstrated a clear and robust delivery of mCherry, highlighting their superior capacity for heterologous protein transport. The OMVs from the ∆*degS* mutant also facilitated the delivery of mCherry into the HT-29 cells, exhibiting fluorescence levels slightly higher than those observed with the WT strain but markedly lower than those achieved with the ∆*tolR* mutant (Figure 6B).

These findings underscore the utility of SP*_ompA_* in targeting mCherry into OMVs and facilitating its delivery into eukaryotic cells. Furthermore, the enhanced delivery efficiency observed in the ∆*tolR* mutant highlights the pivotal role of this hypervesiculatory strain in optimizing protein transport via OMVs under the conditions tested.

### 2.7. Comparative Immunogenic Potential of S. Typhi OMVs and Hypervesiculating Mutants Delivering mCherry as a Model Antigen in Mice

OMVs are emerging as promising platforms for antigen delivery due to their nanoscale size, immunogenic properties, and capacity to encapsulate heterologous proteins [2,9,10]. Incorporating specific antigens into OMVs, facilitated by signal peptides such as SP*_ompA_*, offers a strategy to enhance their biotechnological applications, particularly in vaccine development. However, the immunogenic potential of *S.* Typhi OMVs carrying heterologous proteins must be rigorously evaluated to determine their capacity to elicit specific and robust antibody responses.

In this study, we sought to investigate the immunogenicity of OMVs derived from *Salmonella* Typhi WT and hypervesiculating mutants (∆*tolR* and ∆*degS*) engineered to deliver mCherry, a model heterologous protein. We evaluated the antibody response in BALB/c mice to determine whether hypervesiculating OMVs could enhance antigen-specific immune responses compared to the antigen alone (mCherry) and OMVs from the WT strain.

**Figure 7:**
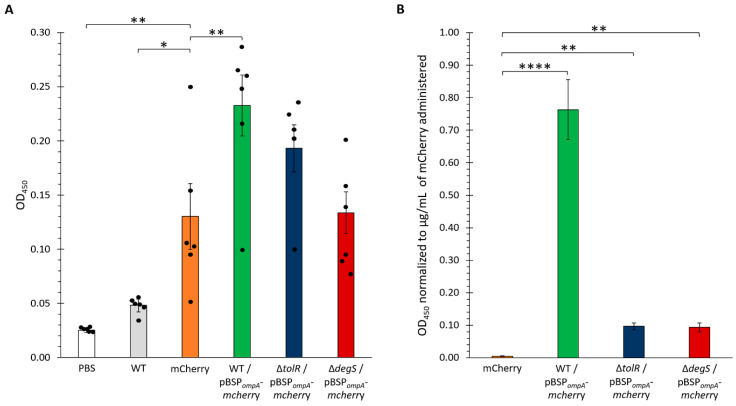
Antibody responses against mCherry following immunization with OMVs and mCherry protein. (**A**) mCherry-specific antibody responses in BALB/c mice. Six groups of mice (n = 6 per group) were immunized intraperitoneally with the following formulations: PBS (vehicle control), OMVs from *S.* Typhi WT without any plasmid (WT group; 25 µg OMVs, quantified protein content), purified mCherry protein as a positive control (25 µg), OMVs from WT *S.* Typhi harboring the pBSP*_ompA_*-*mcherry* plasmid (WT/pBSP*_ompA_*-*mcherry*; 25 µg OMVs, quantified protein content), and OMVs from hypervesiculating ∆*tolR* and ∆*degS* mutants carrying the pBSP*_ompA_*-*mcherry* plasmid (25 µg OMVs for each mutant, quantified protein content). Booster immunizations were administered 14 days after the initial dose. Serum samples were collected two weeks post-booster, and mCherry-specific IgG levels were quantified by ELISA. OD_450_ values represent the mean antibody levels with individual data points displayed. (**B**) Antibody responses normalized to mCherry concentration in OMVs. Fluorescence-based quantification was performed to account for varying mCherry content in the OMVs, and the results were used to normalize antibody responses. A calibration curve was generated to correlate fluorescence intensity with mCherry concentration (Figure S6A). OMVs from ∆*tolR* and ∆*degS* mutants exhibited higher mCherry content per OMV than WT-derived OMVs (Figure S6B). After normalization, WT OMVs harboring pBSP*_ompA_*-*mcherry* induced the highest anti-mCherry antibody responses, followed by ∆*tolR* and ∆*degS* OMVs, which elicited comparable responses but significantly lower than WT-derived OMVs. Purified mCherry alone, despite adjuvant addition, generated minimal antibody levels when normalized to mCherry concentration. Statistical analysis was performed using one-way ANOVA with Tukey′s post hoc test (* *p* < 0.05; ** *p* < 0.01; **** *p* < 0.0001).

To evaluate the immunogenic potential of OMVs loaded with mCherry, six groups of BALB/c mice were immunized with different formulations, including OMVs from *S.* Typhi WT, OMVs from hypervesiculating mutants (∆*tolR* and ∆*degS*), and purified mCherry protein as a positive control. In these experiments, only mCherry alone was adjuvanted with Imject^TM^ Alum (Thermo Scientific, Waltham, MA, USA). Serum samples were collected two weeks post-booster immunization, and the levels of mCherry-specific IgG antibodies were quantified by ELISA (Figure 7A).

The results showed significant differences in the antibody responses among the groups. As expected, the PBS control group (vehicle only) displayed negligible OD_450_ values, serving as a baseline and confirming the lack of a specific immune response against mCherry. Similarly, the mice immunized with OMVs from the WT strain lacking any plasmid (WT group) exhibited low antibody levels, indicating minimal immunogenicity in the absence of a specific antigen. In contrast, the group that received the purified mCherry protein generated a robust antibody response, serving as the positive control.

Notably, the OMVs from the *S.* Typhi WT harboring the pBSP*_ompA_*-*mcherry* plasmid induced significantly higher antibody levels than the negative controls (PBS and WT OMVs), demonstrating the successful delivery and presentation of mCherry via OMVs. Among the groups receiving OMVs loaded with mCherry (WT, ∆*tolR*, and ∆*degS*), the most pronounced responses were observed with the OMVs from the WT and ∆*tolR* strains, which elicited the highest OD_450_ values. The OMVs from the ∆*degS* mutant also induced a robust immune response, though slightly lower than that observed with the WT and ∆*tolR* OMVs. These findings highlight the capacity of OMVs, particularly those from WT and *∆tolR* strains, to efficiently deliver heterologous antigens and stimulate strong antigen-specific immune responses.

Given that the OMVs derived from the WT, ∆*tolR*, and ∆*degS* strains incorporated varying amounts of mCherry, we aimed to standardize the antibody responses observed in the immunized mice to the amount of mCherry delivered. To achieve this, a fluorescence-based calibration curve was generated to correlate fluorescence intensity with mCherry concentration. Serial dilutions of purified mCherry were prepared, and fluorescence measurements were performed using a Synergy H1 microplate reader at excitation and emission wavelengths of 587 and 630 nm, respectively. The resulting calibration curve displayed excellent linearity (R^2^ = 0.999; Figure S6A), allowing for accurate quantification of mCherry in the OMV samples based on their fluorescence values. This approach ensured that the antibody responses elicited in the mice could be normalized to the precise amount of mCherry delivered, facilitating a more robust comparison of the immunogenic potential across the different OMV groups.

We applied the fluorescence-based calibration curve described previously to estimate the concentration of mCherry incorporated into the OMVs used for mouse immunization. By measuring the fluorescence intensity of the OMV samples, we quantified the amount of mCherry in each condition (WT, ∆*tolR*, and ∆*degS* OMVs; Figure S6B). This analysis revealed significant differences in the mCherry content among the strains, with the ∆*tolR* OMVs exhibiting the highest concentration of mCherry, followed by the ∆*degS* OMVs and the WT OMVs, which displayed the lowest levels. These quantified concentrations were subsequently used to normalize the antibody responses elicited in the immunized mice, ensuring a precise comparison of the immunogenic potential across the different OMV groups. The results of the ELISA (Figure 6) showed that the WT-derived OMVs harboring the pBSP*_ompA_*-*mcherry* plasmid elicited the highest OD_450_ values, even after normalization, indicating a significantly stronger anti-mCherry antibody response. In contrast, the OMVs from the ∆*tolR* and ∆*degS* mutants induced markedly lower antibody levels when adjusted for the amount of mCherry delivered, with comparable responses.

These findings indicate that, despite incorporating lower amounts of mCherry, WT OMVs elicit a stronger immune response, characterized by higher antibody titers, compared to OMVs from the hypervesiculating ∆*tolR* and ∆*degS* mutants. Even considering that affinity-purified mCherry was administered with an adjuvant, enhanced immunogenicity suggests that WT OMVs may be more efficient in delivering and presenting antigens to the immune system and in stimulating robust immune activation. This underscores the efficiency of OMVs as a delivery platform, promoting a more effective immune response elicited by mCherry-loaded OMVs (especially *S.* Typhi WT OMVs) compared to the adjuvanted mCherry protein alone.

Moreover, all the OMVs were significantly more effective than purified mCherry alone in inducing anti-mCherry antibody titers, emphasizing their critical role as delivery vehicles. By potentially enhancing antigen stability, promoting uptake by antigen-presenting cells, and/or facilitating effective presentation, OMVs contribute to a more robust immune response. Notably, the superior performance of the WT OMVs further suggests that their unique composition, structural integrity, or immunostimulatory properties maximize immune activation, positioning them as promising platforms for antigen delivery and immune system potentiation.

## 3. Discussion

In this study, we determined whether hypervesiculating *Salmonella* Typhi mutants (∆*tolR* and ∆*degS*) could effectively incorporate and deliver a heterologous protein (mCherry) into eukaryotic cells and evaluated their capacity to elicit immune responses in a murine model. To address the persistent challenge of efficiently packaging foreign proteins into bacterial outer membrane vesicles (OMVs), we employed the *ompA* signal peptide (SP*_ompA_*) to enhance mCherry loading.

Our findings show that the ∆*tolR* mutant stands out for its robust ability to encapsulate mCherry within OMVs and deliver it successfully to human epithelial cells, underscoring its potential as a vehicle for heterologous protein transport. Nevertheless, despite carrying less mCherry, wild-type (WT) OMVs induced a stronger immune response in mice, indicating that the composition or immunostimulatory properties of WT OMVs may be more conducive to eliciting potent immunogenicity. This contrast highlights how hypervesiculation can affect OMV composition, leading to distinct functional phenotypes: ∆*tolR* excels at protein delivery, while WT OMVs appear superior in promoting a robust immune response.

Overall, the results presented here demonstrate that both native and genetically engineered *S.* Typhi OMVs offer versatile, distinct, and promising platforms for heterologous protein delivery and immunological applications. By tailoring genetic modifications to optimize either cargo encapsulation or immunostimulatory potency, *S.* Typhi OMVs provide a flexible framework that could be exploited in developing new biotechnological and therapeutic strategies.

The disruption of *tolR* and *degS* significantly modified OMV biogenesis in *S.* Typhi (Figure 1). Both deletions led to hypervesiculation, but the scale and nature of these changes differed between the two mutations, reflecting the distinct roles of TolR and DegS in outer membrane integrity and envelope stress regulation [13,15,41,43]. Compared to the wild type, the ∆*tolR* mutant exhibited greater OMV output and wider vesicle size variation, consistent with previous evidence showing that compromising the Tol-Pal system intensifies vesiculation [13,15,41]. The ∆*degS* mutant, by contrast, displayed even higher OMV production and increased heterogeneity, aligning with the known influence of σ^E^-mediated stress responses on membrane remodeling [13,15,43]. Notably, the TEM analysis revealed that OMVs from the ∆*tolR* and ∆*degS* mutants were not only larger compared to the WT but also apparently exhibited additional membrane structures indicative of outer–inner membrane vesicles (OIMVs) or multilamellar vesicles (MVs). These complex vesicular forms, previously described in *tol* mutants [17,42], suggest that hypervesiculation in these strains is accompanied by alterations in membrane integrity that promote the encapsulation of cytoplasmic biomolecules. The presence of OIMVs/MVs in the mutants, but not in the WT preparation, suggests a differential cargo profile that may influence the efficiency and specificity of heterologous protein loading and delivery, explaining the presence of mCherry without SP*_ompA_* in Δ*tolR*-derived OMVs.

SDS-PAGE profiles revealed that both the ∆*tolR* and ∆*degS* mutations altered OMV protein composition, consistent with reports that genetic or environmental perturbations can reshape vesicle cargo [13,57]. Despite normalizing the OMV samples by total protein content (30 µg), the SDS-PAGE analysis revealed noticeable differences in band intensities, particularly for ∆*tolR* OMVs. This discrepancy may be partially explained by OMV size heterogeneity and the formation of outer-inner membrane vesicles (OIMVs)/multilamellar vesicles (MVs), which can alter the protein-to-vesicle ratio. Thus, the same total protein load may not correspond to equivalent vesicle numbers or comparable cargo distributions. Moreover, mutations in *tolR* and *degS* can affect cellular physiology, e.g., nutrient uptake and protein translocation [58,59], leading to additional shifts in OMV protein composition. An additional factor to consider is that distinct OMV populations may exhibit varying proportions of specific proteins, particularly those with larger molecular weights. In such cases, highly abundant proteins could dominate the total protein quantification, effectively under-representing less abundant proteins in the SDS-PAGE profile. This phenomenon may further account for the observed variations in band intensities across the different OMV samples despite loading equal amounts (30 µg) of total protein. Collectively, these findings underscore the complexity of OMV biogenesis and composition, highlighting the need to carefully interpret apparent differences in protein profiles and to account for both vesicle heterogeneity and differential cargo loading when designing OMV-based biotechnological applications.

Our investigations confirm that the *S.* Typhi OmpA signal peptide (SP*_ompA_*) efficiently directs heterologous proteins into OMVs, representing a key step for enhancing OMV-based protein delivery systems. While earlier studies noted that not all signal peptides (SPs) function equally well for OMV targeting [31,36], they did not examine *S.* Typhi, especially its hypervesiculating mutants. Given the increased OMV yield of ∆*tolR* and ∆*degS*, validating SP*_ompA_* in these backgrounds was critical for determining scalability and biotechnological potential.

Previous work demonstrated the feasibility of using OmpA to localize proteins within *E. coli* OMVs [53], but its applicability to *S.* Typhi remained untested. Here, we show that SP*_ompA_* remains effective, even under genetically altered vesiculation conditions, in enriching mCherry within OMVs. This finding underlines the importance of empirically testing targeting sequences in various bacterial strains, particularly when significant changes in OMV composition or biogenesis (as in ∆*tolR* and ∆*degS*) might alter cargo loading efficiency.

Figure 2 and Figure 3 collectively demonstrate that SP*_ompA_* is pivotal for directing mCherry into OMVs. The Western blots in Figure 2 show that cytoplasmic mCherry alone (pB*mcherry*) barely appears in ∆*tolR* OMVs and is undetectable in WT or ∆*degS* OMVs. The presence of cytoplasmic mCherry in ∆*tolR* OMV preparations is likely due to the presence of outer–inner membrane vesicles (OIMVs) and multilamellar vesicles (MVs), which can incorporate cytoplasmic proteins since it has been described that *tol* mutants are prone to produce this kind of vesicles [17,42]. In contrast, all the strains exhibit strong mCherry bands when SP*_ompA_* is employed (pBSP*_ompA_*-*mcherry*). It is important to note that different vesicle numbers (i.e., OMVs but also OIMVs and MVs) with inhomogeneous mCherry content were probably loaded on the gels, especially for the Δ*tolR* mutant; thereby, hypervesiculation increases the total number of mCherry OMVs, as corroborated by cytometry. Although the ∆*tolR* mutant consistently shows the highest fluorescence in the OMV fraction (likely resulting from a combination of OIMVs, hypervesiculation, and SP*_ompA_*-mediated targeting), the ∆*degS* mutant also benefits significantly from the signal peptide, demonstrating that different hypervesiculation pathways can be exploited to effectively enhance heterologous protein incorporation into OMVs and ultimately optimize OMV-based delivery systems.

Linking mCherry (a robust fluorescent reporter [13,15,41,43]) to SP*_ompA_* significantly boosted its packaging in the *S.* Typhi OMV fraction, confirming that Sec-dependent targeting preserves protein functionality. While some signal peptides can impair protein folding [60] or render proteins nonfunctional (as with specific GFP variants [61]), SP*_ompA_* did not compromise mCherry fluorescence. This parallels findings where *E. coli* SP*_dsbA_* successfully translocated mCherry into the periplasm without losing function [49]. Moreover, although Tat-dependent systems can facilitate OMV loading (e.g., ~6.5-fold for GFP in *E. coli* [62]), our data, using the same technique (fluorescence readings), show even stronger enhancements, up to 77.9-fold, in *S.* Typhi, particularly in the ∆*degS* mutant. Hence, coupling hypervesiculation with Sec-type signal peptides can substantially optimize OMV-based protein encapsulation.

Figure 4 confirms that mCherry, guided by SP*_ompA_*, localizes to the OMV lumen, where it is shielded from external proteases unless the membrane is disrupted. Similar protection assays in other bacterial species [52,53,62] suggest that OMVs can safeguard their intraluminal cargo. Notably, the ∆*tolR* and ∆*degS* mutants retained this protective advantage, suggesting that excessive vesiculation does not inherently compromise lumen integrity.

Encapsulating cargo proteins in OMVs offers a robust mechanism for boosting protein yields and protecting labile proteins from environmental stress [63,64]. Moreover, such encapsulation allows for more straightforward downstream processing and expanded customization. OMVs can be genetically or chemically modified to further stabilize the cargo and adapt to specialized tasks, such as targeted delivery and immune modulation [63,65]. Consequently, OMV encapsulation represents a flexible platform for diverse applications, from enzyme catalysis to next-generation vaccines.

Figure 5 highlights the utility of flow cytometry for quantitatively assessing OMV heterogeneity and cargo loading in *S.* Typhi WT, ∆*tolR*, and ∆*degS*. Unlike bulk assays (e.g., Western blot), flow cytometry distinguishes individual vesicles based on size (complexity) and fluorescence, revealing which fraction of OMVs carries mCherry. When no signal peptide is present, only ∆*tolR* OMVs exhibit low, detectable fluorescence (~4%). The introduction of SP*_ompA_* substantially increases the fraction of fluorescent OMVs in all strains, with ∆*tolR* (11.6%) and ∆*degS* (7.2%) exceeding WT (2.3%). In essence, the hypervesiculation phenotype increases the total number of mCherry-positive OMVs. In this sense, these data qualitatively corroborate earlier experiments (Figure 2 and Figure 3), collectively supporting the notion that hypervesiculation enhances heterologous cargo loading when paired with a targeting signal.

Although flow cytometry is more commonly applied to eukaryotic extracellular vesicles [66,67], it is equally promising for bacterial OMVs, particularly when specialized procedures (e.g., size exclusion chromatography or TIM4-affinity purification) are used to reduce noise from similarly sized particles [56,68,69,70]. Despite challenges in detecting nanoscale vesicles, ongoing advances in instrument sensitivity and sample preparation continue to expand flow cytometry′s applicability in analyzing bacterial OMVs. By enabling single-particle analysis of OMVs, flow cytometry not only confirms the enhanced mCherry loading observed in the ∆*tolR* and ∆*degS* mutants but also provides quantitative insights into the heterogeneity and cargo distribution within these vesicles. This approach, common in the study of eukaryotic extracellular vesicles, proves equally promising for bacterial OMVs, offering a higher-resolution understanding of their biogenesis and functional capacity, key elements in advancing OMV-based biotechnological and immunological applications.

Figure 6 and Figure S4 fill an important gap by demonstrating, for the first time, to our knowledge, that *S.* Typhi OMVs (including hypervesiculatory mutants) can be endocytosed by human epithelial cells. Although OMV internalization is established in related bacteria such as *E. coli* and *S.* Typhi [54,55,56], *S.* Typhi OMVs have not been similarly scrutinized. Using HT-29 cells, a robust epithelial model for assessing OMV endocytosis [54], we show that OMVs from WT, ∆*tolR*, and ∆*degS* are internalized with minimal cytotoxicity (Figure S5), suggesting that *S.* Typhi OMVs could serve as safe and effective delivery vehicles for heterologous proteins.

Interestingly, while *E. coli* Nissle 1917 ∆*tolR* OMVs reportedly exhibit reduced internalization [16]; we did not observe a marked defect in ∆*tolR*-derived OMVs from *S.* Typhi. Nevertheless, they appeared to stay closer to the cell periphery at early time points (30 min), although our experiments did not quantify uptake kinetics. This underscores how even highly conserved genes like *tolR* can produce divergent OMV phenotypes depending on the bacterial background [71]. Overall, these findings highlight the importance of verifying OMV-based functions in different species and genetic contexts.

Regarding mCherry delivery, we acknowledge that, in Figure 6, the red fluorescence signal (mCherry) does not strictly co-localize with the green DiO-labeled vesicles but instead appears adjacent or in close proximity to them. This observation could be interpreted as a partial or complete release of mCherry from the vesicles, a phenomenon previously noted for *E. coli* OMVs and their associated cargo [72]. However, our experimental design did not specifically address the precise intracellular distribution of mCherry following OMV internalization. Although the images demonstrate that mCherry is delivered to regions near DiO-stained vesicles, we cannot definitively conclude whether mCherry remains encapsulated within OMVs or is partially released.

The IgG antibody responses observed in mice underline the potential of *S.* Typhi OMVs loaded with mCherry as self-adjuvanted vaccine platforms, as proposed [2,9,10]. Although unmodified WT OMVs (without plasmid) elicited negligible antibody production and mCherry alone adjuvanted with Imject^TM^ Alum induced detectable immunogenicity, OMVs carrying SP*_ompA_*-directed mCherry triggered increased anti-mCherry titers. Notably, ∆*tolR* and ∆*degS* OMVs, despite loading more mCherry overall, produced lower antibody levels (after normalization) than WT OMVs. This discrepancy suggests that OMV composition, structure, or adjuvant-like components can be at least as important for immunogenicity as total antigen content. These observations align with the notion that OMV composition and structural integrity, beyond antigen load, play a key role in modulating immunogenicity [73]. Thus, while hypervesiculation boosts antigen loading, it may not guarantee superior immune responses, underscoring the interplay of OMV properties, antigen conformation, and stimulation of host immunity.

These data indicate that *S.* Typhi OMVs can be potent immunogenic carriers for heterologous antigens, primarily when guided by a suitable signal peptide like SP*_ompA_*. Although hypervesiculating mutants encapsulate more antigen, the WT′s OMV composition may be better suited for efficient antigen presentation, leading to heightened immune responses, at least for the antigen tested here. Therefore, future efforts should optimize loading efficiency and immunostimulatory properties to fully harness OMVs in vaccine development. Moreover, studies utilizing more precise vesicle quantification methods (e.g., nanoparticle tracking analysis) will be critical to further refine these qualitative findings.

Compared with other *Salmonella* serovars, *S.* Typhi OMVs possess distinct immunomodulatory features driven by factors such as the Vi capsular antigen, known to dampen Toll-like receptor activation [40], and the bacterium’s unique, human-specific infection strategy [31,36]. These characteristics might yield OMVs particularly suited for both vaccine applications and other biotechnological tools for therapeutic interventions, especially in scenarios demanding low inflammatory profiles (e.g., immunocompromised individuals, older adults, or cancer patients) [74,75,76]. Although typhoid fever remains a global health concern, many aspects of *S.* Typhi OMV biology remain understudied.

The findings of this study establish that *S.* Typhi OMVs can be systematically engineered to enhance heterologous protein cargo loading while retaining immunogenic activity, thereby laying a foundation for advanced biotechnological applications. Hypervesiculation can be harnessed to increase protein delivery (i.e., through the Δ*tolR* mutation), whereas wild-type OMVs appear to offer stronger immunostimulatory capabilities; these insights could be critical for designing next-generation vaccines or immunotherapies. Furthermore, developing manufacturing strategies that exploit the inherent immunomodulatory traits of *S.* Typhi OMVs might improve vaccine safety and efficacy, particularly in at-risk populations. By bridging the fundamental knowledge of *S.* Typhi pathogenesis with practical engineering approaches, this work paves the way for innovative OMV-based platforms capable of targeted protein delivery, vaccine development, and beyond.

## 4. Materials and Methods

### 4.1. Bacterial Strains and Culture Conditions

*Salmonella enterica* subsp. *enterica* serovar Typhi strain STH2370 (*S. Typhi* WT) [77] was obtained from the Infectious Diseases Hospital Lucio Córdova, Chile. The ∆*tolR* and ∆*degS* mutant strains of *S. Typhi* have been previously described [13,15]. Bacterial strains were routinely cultured in Luria–Bertani (LB) medium, composed of Bacto tryptone (10 g/L, Gibco, Thermo Fisher Scientific, Waltham, MA, USA), Bacto yeast extract (5 g/L, Gibco, Thermo Fisher Scientific, Waltham, MA, USA), and NaCl (5 g/L, Winkler, Lampa, Chile) in distilled water, and incubated at 37 °C with shaking to ensure aeration. When necessary, the medium was supplemented with ampicillin (50 µg/mL, AppliChem GmbH, Darmstadt, Germany), gentamicin (12 µg/mL, Merck, Darmstadt, Germany), or X-gal (5-bromo-4-chloro-3-indolyl-β-D-galactopyranoside, 40 µg/mL, Merck, Darmstadt, Germany). Agar (Thermo Fisher Scientific, Waltham, MA, USA) was added at a final concentration of 15 g/L for solid media. To induce protein expression from the *P_araBAD_* promoter, the cultures were supplemented with 1.33 µmol/L L-arabinose or 1.11 µmol/L D-glucose (Merck, Darmstadt, Germany).

### 4.2. OMV Purification

OMVs were isolated following established protocols with modifications [13,78]. *S.* Typhi strains were cultured in LB medium supplemented with gentamicin and L-arabinose at 37 °C with shaking until reaching an OD_600_ of 1.1. The cultures were centrifuged at 5400× *g* for 10 min at 4 °C to pellet the cells. The supernatant was collected, filtered through a 0.45 µm membrane (0.45 μm, Nalgene^TM^, Gibco, Thermo Fisher Scientific, Waltham, MA, USA), and subsequently ultrafiltered using Ultracel^TM^ 100 kDa ultrafiltration discs (Amicon^TM^ Bioseparations; Merck, Darmstadt, Germany). The filtered supernatant was then ultracentrifuged (Thermo Fisher Scientific Sorvall™ WX, Rotor AH-629, Waltham, MA, USA) at 150,000× *g* for 3 h at 4 °C. After ultracentrifugation, the supernatant was discarded, and the OMV-containing pellet was resuspended in 1 mL of physiological saline solution (0.9% NaCl). The OMVs were stored at −80 °C until further use. The protein concentration was measured using a Pierce™ BCA Protein Assay Kit (Thermo Fisher Scientific, Waltham, MA, USA), while lipid quantification was performed using an FM4-64 fluorescent probe (Thermo Fisher Scientific, Waltham, MA, USA), as previously described [13].

### 4.3. Transmission Electron Microscopy (TEM) and OMV Size Measurement

OMV samples were adsorbed onto formvar-coated slot grids (EMS, Hatfield, PA, USA) and stained with 1% aqueous uranyl acetate (EMS, Hatfield, PA, USA) for 1 min. Following previously described protocols, the grids were examined using a Philips Tecnai 12 (Biotwin, Thermo Fisher Scientific, Waltham, MA, USA) transmission electron microscope [13]. OMV size was determined based on TEM images (3 fields) per biological triplicate, as described in [79,80], and is reported as the vesicle diameter.

### 4.4. SP_ompA_-Mcherry-FLAG Gene Fusion Construction and Cloning

The SP*_ompA_*-*mcherry*-FLAG (called SP*_ompA_*-*mcherry*) gene fusion, consisting of the *S.* Typhi *ompA* signal peptide (21 residues: MKKTAIAIAVALAGFATVAQA) appended to the N-terminus of mCherry, was constructed using overlap extension PCR, as previously described [81,82]. Briefly, the coding sequences (CDSs) of SP*_ompA_* (amplified using primers F SP.ompA + EcoRI and R SP.ompA + mCherry, Table 1) and mCherry (amplified using primers F mcherry + SP.ompA and R mcherry + FLAG + XbaI, Table 1) were obtained by PCR. The templates used were *S.* Typhi genomic DNA for SP*_ompA_* and the plasmid pFCcGi (Addgene #59324, Watertown, MA, USA) for mCherry. The PCR products were purified with an EZNA Gel Extraction Kit (Omega Bio-Tek, Norcross, GA, USA). The gene fusion was created in two steps: (1) Overlap Step. To generate the SP*_ompA_*-mCherry fusion, an initial PCR reaction was performed without primers. The reaction mixture contained 1× PCR master mix and 100 ng of each fragment (i.e., SP*_ompA_* and mCherry). The amplification protocol included an initial denaturation step at 94 °C for 3 min, followed by 15 cycles of denaturation at 94 °C for 45 s, annealing at 72 °C (decreasing 0.5 °C per cycle) for 30 s, and extension at 72 °C for 90 s. The reaction concluded with a final extension step at 72 °C for 10 min. (2) Extension Step: After the overlap reaction, 1 µM of forward and reverse primers (F SP.ompA + EcoRI + R mcherry + FLAG + XbaI, Table 1) was added to amplify the fused product. Amplification conditions included an initial denaturation at 94 °C for 3 min, followed by 30 cycles of denaturation at 94 °C for 45 s, annealing at 60.5 °C (decreasing 0.3 °C per cycle) for 30 s, and extension at 72 °C for 90 s. The reaction ended with a final extension at 72 °C for 10 min.

To generate *mcherry*-FLAG (called *mcherry*) without the signal peptide, the *mcherry* coding sequence was amplified by PCR using the primers F mCherry + EcoRI and R mCherry + FLAG + XbaI. The plasmid pFCcGi (Addgene #59324, Watertown, MA, USA) served as the template for this reaction.

The FLAG coding sequence was added to the reverse primer during amplification to generate SP*_ompA_*-*mcherry* or *mcherry* tagged with a FLAG epitope at the C-terminus.

Both the pBAD33-GM vector (Addgene #65098, Watertown, MA, USA) and the PCR products, either the fused SP*_ompA_*-*mcherry* or *mcherry* alone, were digested with *EcoRI* and *XbaI* restriction enzymes (Promega, Madison, WI, USA) by incubation at 37 °C for 1 h. The digested DNA fragments were purified using an EZNA Gel Extraction Kit (Omega Bio-Tek, Norcross, GA, USA). Ligation reactions were performed using T4 DNA ligase (Promega, Madison, WI, USA) by incubating the digested vector with either the SP*_ompA_*-*mcherry* fusion product or the *mcherry* PCR product at 15 °C for 18 h, resulting in the pBAD-SP*_ompA_*-*mcherry* (pBSP*_ompA_*-*mcherry*) and pBAD-*mcherry* (pB*mcherry*) constructs, respectively. The resulting plasmids were sequenced at Pontificia Universidad Católica de Chile using an ABI PRISM 3500xl Genetic Analyzer (Applied Biosystems, Foster City, CA, USA) with forward and reverse pBAD primers (F pBAD and R pBAD, Table 1). The sequence data, obtained in GenBank format, were analyzed using SnapGene v3.2.1 software (GSL Biotech LLC, Chicago, IL, USA) to confirm the correct construction of plasmids through multiple alignments.

### 4.5. SDS-PAGE

SDS-PAGE was performed as described previously [13]. OMVs, corresponding to 30 µg of protein, were resolved on SDS-PAGE gels and stained with One-Step Blue Protein Gel Stain (Biotium, Fremont, CA, USA) for visualization.

### 4.6. Western Blot Assays

OMVs (30 µg of protein) were resolved by SDS-PAGE and transferred to nitrocellulose membranes. To confirm protein loading, the membranes were stained with Ponceau S (Thermo Fisher Scientific, Waltham, MA, USA), as previously described [83], and subsequently washed with distilled water. For FLAG detection, the membranes were incubated with a primary mouse anti-FLAG M2 monoclonal antibody (1:5000, Sigma-Aldrich, St. Louis, MO, USA), followed by a secondary horseradish peroxidase (HRP)-conjugated goat anti-mouse IgG (1:5000, Sigma-Aldrich, St. Louis, MA, USA). As a control, the membranes were stripped and probed with primary polyclonal antibodies obtained from the serum of mice immunized with *S.* Typhi OMVs (1:1000), followed by the same secondary HRP-conjugated goat anti-mouse IgG (1:5000, Sigma-Aldrich, St. Louis, MA, USA). Protein detection in all cases was performed using enhanced chemiluminescence (Clarity Western ECL Substrate, Bio-Rad, Hercules, CA, USA).

### 4.7. Fluorescence Activity Assay for mCherry in OMVs

The fluorescence activity of mCherry in OMVs was measured using a Synergy H1M microplate reader (BioTek, Winooski, VT, USA). For endpoint fluorescence readings (Figure 3), the excitation wavelength was set to 587 nm, the optimal value for mCherry excitation, while the emission wavelength was recorded at 630 nm. Although the optimal emission wavelength for mCherry is 610 nm, it was adjusted to 630 nm due to the spectral overlap limitations of the plate reader.

### 4.8. Proteinase K Protection Assay

To verify that mCherry was localized within the lumen of OMVs, 30 µg of OMVs (quantified by protein content) were treated with 0.5 µg/mL of proteinase K (Thermo Fisher Scientific, Waltham, MA, USA) at 37 °C for 30 min, either in the presence or absence of 1% SDS (Winkler, Lampa, Chile). The presence of mCherry in the proteinase K-treated OMVs was evaluated using Western blotting, as previously described [52,53], and fluorescence measurements. The OMVs were treated with 2 µg/mL of proteinase K (Thermo Fisher Scientific, Waltham, MA, USA) under the same conditions for fluorescence measurements.

### 4.9. Fluorescent Labeling of OMVs with DiO

OMVs were fluorescently labeled with 1% (*v*/*v*) 3,3′-dioctadecyloxacarbocyanine perchlorate (DiO; Thermo Fisher Scientific, Waltham, MA, USA) by incubation at 37 °C for 20 min, as previously described [84]. Excess dye was removed by washing with phosphate-buffered saline (PBS, Gibco, Thermo Fisher Scientific, Waltham, MA, USA) using a 100 kDa molecular weight cutoff (MWCO) filtration column (Amicon^TM^ Merck, Darmstadt, Germany).

### 4.10. Flow Cytometry Analysis

Flow cytometry was performed following previously described protocols [85]. DiO-labeled OMVs were analyzed using the small particle detector (SP D) of the FACSymphony™ A1 flow cytometer (BD Biosciences, Franklin Lakes, NJ, USA). Fluorescence for the DiO-labeled OMVs was assessed with a 488 laser and detected in the BB-515 channel to establish 100% fluorescence reference levels and analyze particle complexity. On the other hand, the mCherry-positive OMVs were assessed with a 561 nm laser and detected in the PE-Texas red channel. All measurements followed the manufacturer′s guidelines. The control samples included phosphate-buffered saline (PBS; Gibco, Thermo Fisher Scientific, Waltham, MA, USA) alone and PBS mixed with DiO (without OMVs) to set the SP-SSC-A threshold alongside a positive control of DiO-stained OMVs without mCherry. During acquisition, 50,000–100,000 events were recorded per sample. Data were processed and analyzed using FlowJo software (version 10).

### 4.11. Epithelial Cell Culture

The human colorectal adenocarcinoma cell line HT-29 (ECACC 91072201, European Collection of Authenticated Cell Cultures) was cultured in Dulbecco′s Modified Eagle Medium (DMEM, High Glucose, Gibco, Thermo Fisher Scientific, Waltham, MA, USA) containing 4.5 g/L D-glucose, L-glutamine, and 110 mg/L sodium pyruvate. The medium was supplemented with 10% (*v*/*v*) fetal bovine serum (FBS) (Gibco, Thermo Fisher Scientific, Waltham, MA, USA) and 1× antibiotic–antimycotic (Gibco, Thermo Fisher Scientific, MA, Waltham, USA). The cells were maintained at 37 °C in a humidified atmosphere with 5% CO_2_. The culture medium was refreshed every 3–4 days, and the cells were subcultured weekly using 0.25% trypsin–EDTA (0.53 mM) (Gibco, Thermo Fisher Scientific, Waltham, MA, USA).

### 4.12. Confocal Microscopy Analysis of HT-29 Cells Exposed to S. Typhi OMVs Labeled with DiO

HT-29 cells (ECCAC 91072201) were cultured on glass coverslips in a 24-well plate. The cells were seeded at a density of 2 × 10^5^ cells per well and incubated overnight at 37 °C with 5% CO_2_ to allow adherence and initial growth. After incubation, the cells were washed three times with phosphate-buffered saline (PBS, Gibco, Thermo Fisher Scientific, Waltham, MA, USA) to remove the residual medium. They were then treated with OMVs (100 µg/mL quantified by protein content) isolated from *S.* Typhi WT or mutant derivatives, which had been labeled with DiO (Thermo Fisher Scientific, Waltham, MA, USA). The cells were incubated with the OMVs for 30 min or 3 h at 37 °C, followed by thorough PBS washes to remove any unbound OMVs or dye. To preserve the cellular structures, the cells were fixed with 4% paraformaldehyde (PFA, Merck, Darmstadt, Germany) for 20 min at room temperature and washed thrice with PBS. The nuclei were stained with Hoechst 33342 (Thermo Fisher Scientific, Waltham, MA, USA) at 3 µg/mL for 15 min. When needed, the plasma membrane was stained using Alexa Fluor^®^555-conjugated wheat germ agglutinin (WGA, Thermo Fisher Scientific, Waltham, MA, USA) at 5 μg/mL for 15 min. Finally, the coverslips were mounted onto glass slides using Fluoromount-G mounting medium (Thermo Fisher Scientific, Waltham, MA, USA) to stabilize the samples and preserve fluorescence. Imaging was performed using a Leica TCS SP8 confocal microscope (Leica Microsystems, Wetzlar, Germany) loaded with 405 nm (for Hoechst), 488 nm (for DiO), and 552 nm (for Alexa Fluor^®^ 555 and mCherry) lasers. The photomultiplier tube (PMT) detection range was set to 410–483 nm for the Hoechst channel, 505–547 nm for the DiO channel, and 587–726 nm for the WGA or mCherry channel. Confocal images were acquired and processed using LasX (version 3.1.5.16308) and Fiji software (version 2.14) for subsequent analysis. The quantification of mCherry fluorescence intensity (red pixels) in the confocal microscopy images was performed using Fiji software (National Institutes of Health, Bethesda, MD, USA). The data were averaged across a minimum of 10 fields of view per condition, and the results were expressed as mean red pixels per cell. All settings and thresholds were kept consistent throughout the analysis to ensure comparability.

### 4.13. Cell Viability Assay

HT-29 cells (ECCAC 91072201) were seeded at a density of 2 × 10^5^ cells per well in a 24-well plate and cultured overnight at 37 °C in a humidified incubator with 5% CO_2_. Cell viability was evaluated following treatment with OMVs at 100 µg/mL (determined by protein content) derived from *Salmonella* Typhi WT or mutant strains. The cells were exposed to OMVs for 30 min and 3 h. Post-incubation, the cells were harvested by washing twice with phosphate-buffered saline (PBS; Gibco, Thermo Fisher Scientific, Waltham, MA, USA) to remove residual OMVs and detaching them with 0.25% trypsin-EDTA (Gibco, Thermo Fisher Scientific, Waltham, MA, USA). The detached cells were collected by centrifugation at 200× *g* for 7 min, resuspended in 200 µL of PBS, and stained with 0.5 µL of propidium iodide (PI, 1 mg/mL; Sigma-Aldrich, St. Louis, MO, USA). Fluorescence intensities were analyzed using a FACSAria III flow cytometer (Becton Dickinson, San Jose, CA, USA) to determine the proportion of PI-positive cells indicative of compromised cell viability. Data acquisition and processing were conducted using FACSDiva software (version 8.0.2, Becton Dickinson, San Jose, CA, USA), ensuring precise quantification and reproducibility across experimental conditions. To ensure robust measurements, fluorescence readings were obtained after analyzing between 25,000 and 200,000 events per sample.

### 4.14. Immunization and Serum Collection

Female BALB/c mice (6–8 weeks old) were obtained from the Facultad de Odontología, Universidad de Chile (Santiago, Chile). The mice were divided into groups of six and immunized intraperitoneally with 25 µg of OMVs (quantified by protein content) without adjuvant or 25 µg of purified mCherry adjuvanted with 1 mg of Imject^TM^ Alum (Thermo Scientific, Rockford, IL, USA). All preparations were suspended in 100 µL of phosphate-buffered saline (PBS; Gibco, Thermo Scientific, Rockford, USA). OMVs were isolated from *S.* Typhi WT and the ∆*tolR* and ∆*degS* mutants harboring the plasmid pBSP*_ompA_*-*mcherry* previously cultured with L-arabinose. The negative control groups included mice that received 100 µL of PBS with alum alone or OMVs from *S.* Typhi WT lacking any plasmid. As a positive control, a group of mice was administered purified mCherry protein (25 µg) adjuvanted with Alum. Booster immunizations were administered 14 days after the initial immunization. Blood samples were collected via the facial vein two weeks following the booster. The serum was separated by centrifugation at 1500× *g* for 15 min at room temperature, aliquoted, and stored at −80 °C until use. All procedures were approved by the Animal Ethics Committee of Universidad Andrés Bello, Chile, and followed institutional and national guidelines for the care and use of laboratory animals.

### 4.15. Expression of Recombinant mCherry

The plasmid pRSETB-mCherry (Addgene #108857, Watertown, MA, USA) encodes mCherry-6×His and was transformed into *Escherichia coli* BL21-Gold (DE3) cells (Thermo Fisher Scientific, Waltham, MA, USA) for protein expression [86]. Transformants were selected on LB agar plates supplemented with ampicillin (100 µg/mL) and confirmed via colony PCR and sequencing. For protein expression, a single colony was used to inoculate 2 mL of LB medium containing ampicillin (50 µg/mL) and grown overnight at 37 °C with shaking. The overnight culture was then diluted 1:100 into 40 mL of fresh LB medium with ampicillin and incubated at 37 °C for 6 h. Protein expression was induced by adding 1 mg/mL of glucose, and the culture was incubated for an additional 12 h at 30 °C with shaking.

### 4.16. Purification of mCherry

After induction, to purify recombinant mCherry-6 × His, the intact cells were pelleted using a Beckman Coulter centrifuge (Brea, CA, USA) at 9000× *g* for 14 min at 4 °C. The cell pellet was resuspended in lysis buffer (50 mM Tris-HCl, pH 8.0, 300 mM NaCl, 10 mM imidazole, 1 mg/mL lysozyme, and 1× protease inhibitor cocktail; Sigma-Aldrich, St. Louis, MO, USA) and incubated on ice for 30 min. The cells were lysed by sonication on ice (20 cycles of 30 s on/30 s off at 50% amplitude using a probe sonicator; Branson Ultrasonics, Danbury, CT, USA). The lysate was clarified by centrifugation at 12,000× *g* for 30 min at 4 °C. The supernatant containing soluble mCherry-6 × His was applied to a nickel–nitrilotriacetic acid (Ni-NTA) affinity column (HisTrap HP, Cytiva, Marlborough, MA, USA) pre-equilibrated with equilibration buffer (50 mM Tris-HCl, pH 8.0, 300 mM NaCl, and 10 mM imidazole). Unbound proteins were removed by washing the column with 10 volumes of wash buffer (50 mM Tris-HCl, pH 8.0, 300 mM NaCl, and 20 mM imidazole). mCherry-6 × His was eluted with elution buffer containing 250 mM imidazole in 50 mM Tris-HCl, pH 8.0, and 300 mM NaCl. To further concentrate mCherry-6 × His and remove imidazole, the eluted protein fractions were pooled and concentrated using Amicon Ultra-15 centrifugal filter units (10 kDa molecular weight cutoff; Merck, Darmstadt, Germany). Fractions corresponding to mCherry-6 × His were collected and analyzed by SDS-PAGE to confirm purity. Additionally, fluorescence measurements were performed using a Synergy H1 microplate reader (BioTek, Winooski, VT, USA) with excitation and emission wavelengths set at 587 nm and 630 nm, respectively. The concentration of purified mCherry was determined using a Pierce™ BCA Protein Assay Kit (Thermo Fisher Scientific, Waltham, MA, USA). It is worth noting that this protocol may feature trace amounts of immunogenic contaminants derived from *E. coli* cell lysates. The protein was aliquoted and stored at −80 °C until use.

### 4.17. Enzyme-Linked Immunosorbent Assay (ELISA)

ELISA was performed to quantify antibodies in mouse serum that recognized the mCherry protein. Ninety-six well plates were coated overnight at 4 °C with 100 µL per well of purified mCherry-6 × His protein (2 µg/mL) in carbonate–bicarbonate buffer (pH 9.6). After coating, the wells were washed three times with phosphate-buffered saline (PBS; Gibco, Thermo Fisher Scientific, Waltham, MA, USA) containing 0.05% Tween-20 (PBST) and blocked with 5% bovine serum albumin (BSA, Sigma-Aldrich, St. Louis, MO, USA) in PBS for 1 h at 37 °C. Serum samples were diluted 1:100 in PBS containing 1% BSA and added to the wells (100 µL per well). The plates were incubated for 2 h at 37 °C, followed by three washes with PBST. Bound antibodies were detected using a horseradish peroxidase (HRP)-conjugated goat anti-mouse IgG secondary antibody (1:5000, Thermo Fisher Scientific, Waltham, MA, USA), incubated for 1 h at 37 °C. Following incubation, the wells were washed three times with PBST and developed with 100 µL of 3,3′,5,5′-tetramethylbenzidine (TMB) substrate solution (Thermo Fisher Scientific, Waltham, MA, USA). The reaction was stopped by adding 50 µL of 1 M sulfuric acid, and the optical density (OD) at 450 nm was measured using a Synergy H1 microplate reader (BioTek, Winooski, VT, USA).

### 4.18. Fluorescence-Based Calibration Curve for mCherry Quantification

A fluorescence-based calibration curve was generated to estimate the concentration of mCherry in the samples using its fluorescence intensity. Purified mCherry was prepared at an initial concentration of 50 µg/mL in phosphate-buffered saline (PBS; Gibco, Thermo Fisher Scientific, Waltham, MA, USA). Fluorescence measurements were performed using a Synergy H1 microplate reader (BioTek, Winooski, VT, USA) with excitation and emission wavelengths set at 587 and 630 nm, respectively. Serial two-fold dilutions of the initial mCherry solution were prepared in PBS, resulting in concentrations ranging from 50 µg/mL to 0.024 µg/mL. Each dilution (200 µL) was added to a 96-well black microplate and measured in quadruplicate to ensure accuracy and reproducibility. Fluorescence readings, expressed in arbitrary fluorescence units (AFUs), were plotted against the known mCherry concentrations on a logarithmic scale. A linear regression analysis was performed to generate the calibration curve, yielding a standard equation (y = 2550.5x, R^2^ = 0.999), subsequently used to estimate the concentration of mCherry in the experimental samples based on their fluorescence intensity.

## 5. Conclusions

Our study demonstrates that *Salmonella* Typhi OMVs can be engineered to encapsulate and deliver a heterologous protein (mCherry) via a Sec-dependent mechanism mediated by the OmpA signal peptide (SP*ompA*). Deletion of *tolR* and *degS* yields hypervesiculating strains that produce heterogeneous OMV populations, which increases the overall number of mCherry-positive vesicles. Notably, while the ∆*tolR* mutant exhibited enhanced mCherry delivery to epithelial cells, the WT OMVs elicited a more robust anti-mCherry immune response in the mice after normalization for antigen content. These findings underscore that an elevated antigen load does not necessarily correlate with improved immunogenicity and that OMV composition and structural integrity are critical determinants of functional outcomes. Although our quantitative comparisons are limited by normalization to total protein and inherent vesicle heterogeneity, the data consistently indicate that *S.* Typhi OMVs, when properly engineered, represent a promising platform for heterologous protein delivery and vaccine applications.

## Figures and Tables

**Table 1:** Primer sequences used in this study.

Primer	Sequence
F SP.ompA + EcoRI	5′AATTGAATTCATGAAAAAGACAGCTATCGCG
R SP.ompA + mcherry	5′TATCCTCCTCGCCCTTGCTCACCATGGCCTGCGCTACGGTAGCGA
F mcherry + SP.ompA	5′TGGTTTCGCTACCGTAGCGCAGGCCATGGTGAGCAAGGGCGAGGA
R mcherry + FLAG + XbaI	5′CTAGTCTAGATTACTTGTCGTCATCGTCTTTGTAGTCCTTGTACAGCTCGTCCATGC
F mcherry + EcoRI	5′AATTGAATTCATGGTGAGCAAGGGCGAGGA
F pBAD	5′ATGCCATAGCATTTTTATCC
R pBAD	5′GATTTAATCTGTATCAGG
tolR-N	5′ TGGAAACGCTGGAAACGCAT
tolR-C	5′CTGGCTTGGCGGTTTAGGAAT
degS-N	5′TGCCGAACTGCGTTCACGTAT
degS-C	5′AGACGTCGGCACCGAATGTT

## Data Availability

The original contributions presented in this study are included in this article/the Supplementary Materials. Further inquiries can be directed to the corresponding author(s).

## Supplementary Materials

The following supporting information can be downloaded at https://www.mdpi.com/article/10.3390/ijms26094223/s1.
