# Supplementary material for: Hypervesiculation Meets Sec-Targeting: Enhancing Heterologous Protein Loading in Salmonella Typhi Outer Membrane Vesicles for Delivery and Immune Response
Source: Int J Mol Sci. 2025 Apr 29;26(9):4223. doi: 10.3390/ijms26094223 (PMC12072155; doi:10.3390/ijms26094223)
Supplement: Supplementary file 1 [file ijms-26-04223-s001.zip › ijms-3520516-supplementary.pdf]

Supplementary material

Article

Hypervesiculation Meets Sec-Targeting: Enhancing Heterologous Protein Loading in *Salmonella* Typhi Outer Membrane Vesicles for Delivery and Immune Response

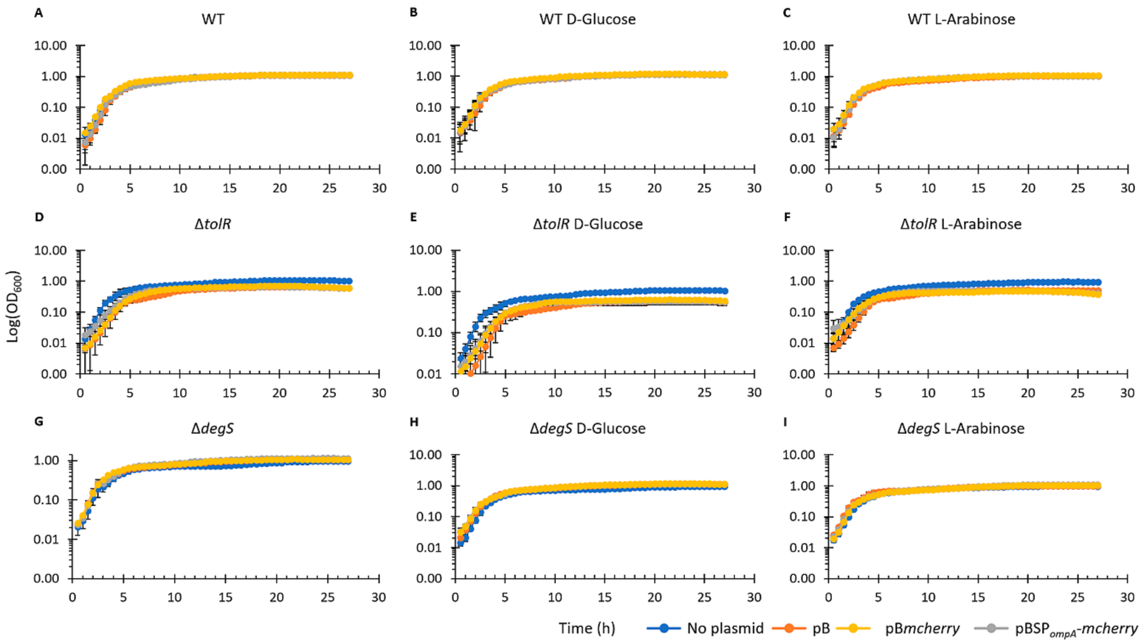

Figure S 1. Growth curves of *Salmonella* Typhi WT, Δ*tolR*, and Δ*degS* strains under different growth conditions and plasmid constructs. Growth was monitored as OD_{600} over time in LB medium (Panels A, D, G), LB + D-glucose (Panels B, E, H), and LB + L-arabinose (Panels C, F, I). Panels A–C correspond to the *S.* Typhi WT strain, Panels D–F to the *S.* Typhi Δ*tolR* mutant, and Panels G–I to the *S.* Typhi Δ*degS* mutant. For each panel, growth was assessed for the strain without a plasmid (blue), with an empty plasmid (pB, corresponding to pBAD33-GM, orange), with a plasmid carrying cytoplasmic mCherry cloned into pBAD33-GM under the P_{araBAD} promoter (pB*mcherry*, yellow), or with the *S.* Typhi OmpA signal peptide fused to the N-terminus of mCherry cloned into pBAD33-GM under the P_{araBAD} promoter(pSP$_{ompA}$-mcherry, gray).

Growth dynamics of *S.* Typhi WT, Δ*tolR*, and Δ*degS* strains were analyzed under different conditions to assess the effects of L-arabinose, D-glucose, and plasmid carriage. Optical density at 600 nm (OD_{600}) was monitored over time, revealing key differences in growth and maximum OD_{600} values between strain backgrounds and experimental conditions.

Under all tested conditions, neither the supplementation of L-arabinose nor the combination of L-arabinose and D-glucose significantly influenced the growth of WT, Δ*tolR*, or Δ*degS* strains. WT cells grew comparably in LB (Panel A), LB + D-glucose (Panel B), and LB + L-arabinose (Panel C), indicating metabolic resilience to these supplements. Similarly, the Δ*tolR* (Panels D–F) and Δ*degS* (Panels G–I) mutants exhibited no measurable changes in growth across these conditions, suggesting that neither sugar supplementation altered their growth dynamics.

When comparing strains, the WT strain consistently demonstrated the highest growth and maximum OD_{600}, regardless of condition. In contrast, the Δ*degS* mutant exhibited slightly slower growth, reflecting a minor growth defect likely associated with envelope stress due to the loss of DegS. The Δ*tolR* mutant displayed the most pronounced growth impairment, characterized by a slower growth rate and significantly lower maximum OD_{600} values compared to WT and Δ*degS*, consistent with membrane instability caused by the deletion of *tolR*.

Plasmid carriage had varying effects depending on the strain background. In the WT strain (Panels A–C), plasmid presence, whether empty (pB, control) or encoding mCherry (pB*mcherry*) or SP_{ompA}-mCherry (pB SP_{ompA}-*mcherry*), did not affect growth rates or maximum OD_{600}, highlighting the metabolic robustness of WT cells. Similarly, in the Δ*degS* mutant (Panels G–I), plasmid-bearing strains grew comparably to the plasmid-free control, suggesting that the additional metabolic burden of plasmid maintenance and recombinant protein expression was well-tolerated. In contrast, the Δ*tolR* mutant (Panels D–F) showed growth defects associated with plasmid carriage. Strains bearing plasmids exhibited slower growth and lower maximum OD_{600} than the plasmid-free strain, indicating that plasmid maintenance exacerbates the inherent growth defect caused by membrane instability in this mutant.

Despite the observed growth defects, all strains, including the mutants, grow sufficiently to produce OMVs. Notably, the Δ*tolR* and Δ*degS* mutants exhibit hypervesiculation, generating high quantities of OMVs that compensate for any growth impairments caused by the mutations (Figure 1). This makes both mutant strains reliable and efficient sources of OMVs, even when carrying plasmids encoding fusion proteins.

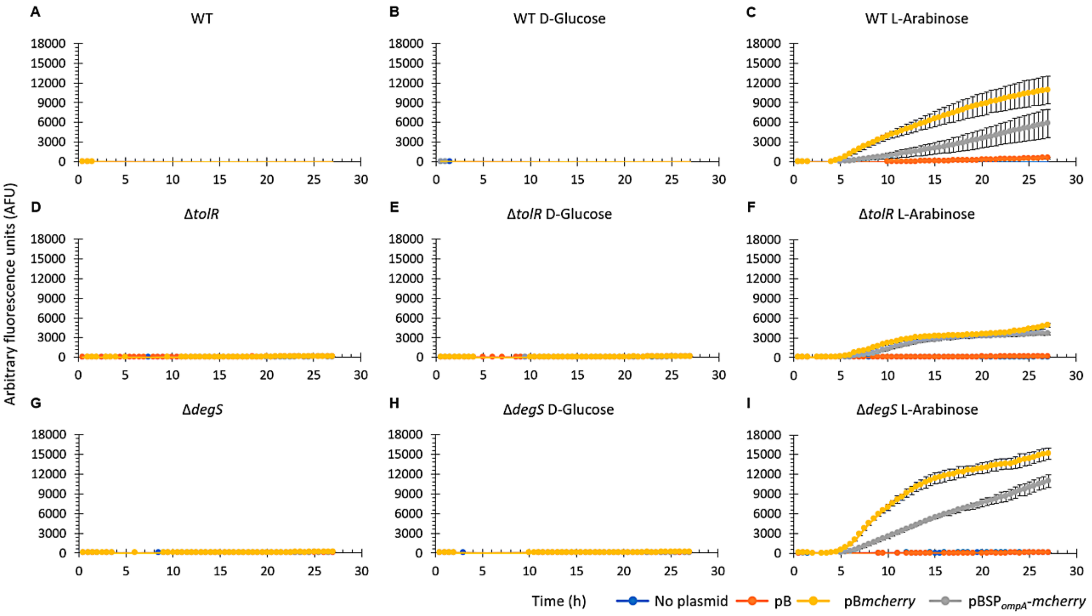

Figure S 2. Fluorescence analysis of mCherry in *Salmonella* Typhi WT, Δ*tolR*, and Δ*degS* strains under diverse growth conditions and plasmid constructs. The fluorescence of mCherry (excitation: 587 nm; emission: 630 nm) was evaluated in strains under various growth conditions and plasmid configurations. Measurements of mCherry fluorescence were conducted over time in LB medium (Panels A, D, G), LB supplemented with D-glucose (Panels B, E, H), and LB supplemented with L-arabinose (Panels C, F, I). Panels A–C represent data from the WT strain, Panels D–F correspond to the Δ*tolR* mutant, and Panels G–I to the Δ*degS* mutant. For each panel, mCherry fluorescence was assessed for the strain without a plasmid (blue), with an empty plasmid (pB, corresponding to pBAD33-GM, orange), with a plasmid carrying cytoplasmic mCherry cloned into pBAD33-GM under the P_{araBAD} promoter (pB*mcherry*, yellow), or with the *S.* Typhi OmpA signal peptide fused to the N-terminus of mCherry cloned into pBAD33-GM under the P_{araBAD} promoter(pSP$_{ompA}$-mcherry, gray).

No fluorescence was detected in any strain grown in LB or LB supplemented with D-glucose. This included strains harboring either the pB*mcherry* or pBSP$_{ompA}$-*mcherry* plasmids, as expected. Fluorescence was observed only in the presence of L-arabinose in strains containing either pB*mcherry* or pBSP$_{ompA}$-*mcherry*, and fluorescence positively correlated with bacterial growth (see Figure S 1). As expected, strains without a plasmid or carrying the empty plasmid (pB, corresponding to pBAD33-GM) showed no fluorescence. In the *S.* Typhi WT and Δ*degS* strains, fluorescence was higher in strains carrying pB*mcherry* than those with pBSP$_{ompA}$-*mcherry*. This difference may be attributed to the presence of the OmpA signal peptide and subsequent translocation to the periplasm, potentially affecting mCherry folding. In the *S.* Typhi Δ*tolR* strain, no significant difference in fluorescence was observed between pB*mcherry* and pBSP$_{ompA}$-*mcherry* constructs. Additionally, *S.* Typhi Δ*tolR* exhibited the lowest fluorescence levels compared to other strains, which correlated with reduced growth of this strain (see see Figure S 1).

Despite the observed fluorescence variations and growth differences among the strains, the mutant and constructs are well-suited for the study's objectives. The arabinose-induced fluorescence confirms the functionality of the constructs, while the differential signal intensities between strains provide insights into the effects of specific mutations on the expression and functionality (assessed as fluorescence) of the reporter mCherry.

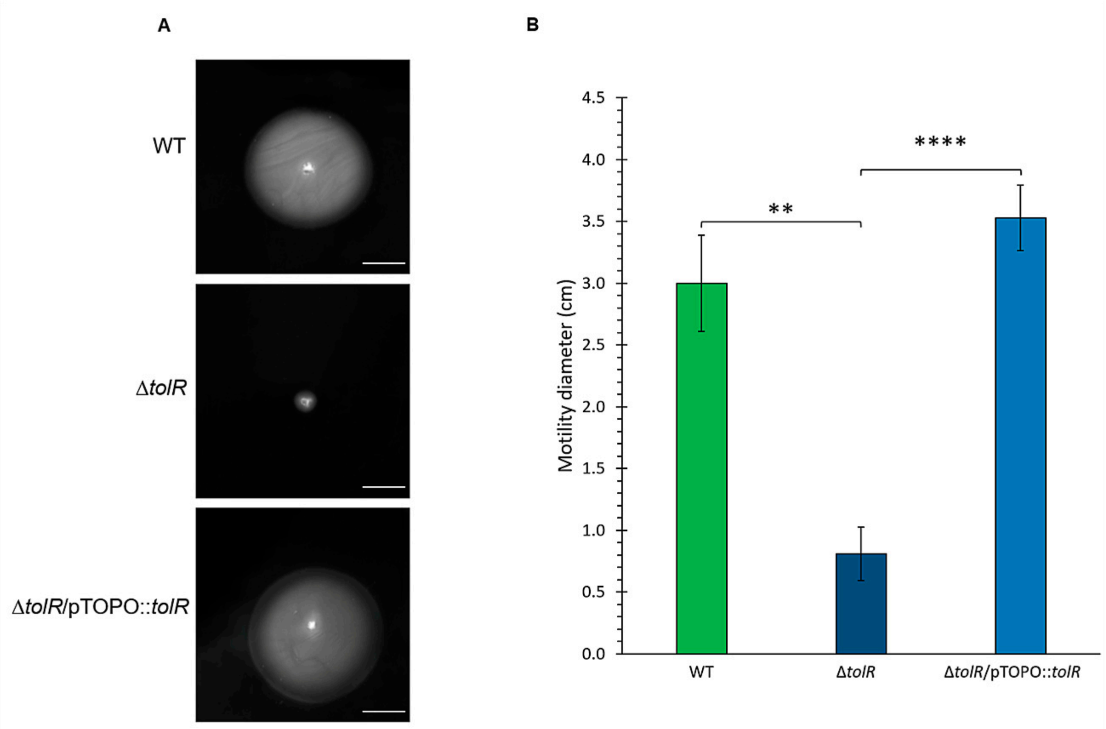

Figure S 3. Motility assay (swimming) in the agar of *S.* Typhi WT, Δ*tolR*, and Δ*tolR*/pTOPO::*tolR* (complemented strain) **(A)** Visual representation of motility halos in 0.3% semisolid agar after 16 h of incubation at 37°C. The WT strain exhibits characteristic motility halos indicative of a flagellated and motile bacterium. In contrast, the *S.* Typhi Δ*tolR* strain shows a significant reduction in motility, evidenced by the smaller halo. The *S.* Typhi Δ*tolR* complemented strain (*S.* Typhi Δ*tolR*/pTOPO::*tolR*) restores motility, displaying halos comparable in size to the WT strain, confirming that the motility defect in Δ*tolR* is solely due to the absence of *tolR*. Bars represent 1 cm. **(B)** Quantification of motility based on halo diameter (cm) in semisolid agar. Data represent the mean ± standard error of three independent experiments. Significant differences in motility were observed between the WT and Δ*tolR* strains ($p < 0.01$), whereas no significant differences were detected between the WT and the complemented strain (one-way ANOVA followed by Tukey's post hoc test).

The *tolR* gene, including its native promoter region, was amplified by PCR from the genomic DNA of *S.* Typhi WT using specific primers designed to flank the gene (Table 1). The amplified product was ligated into the pCR®2.1-TOPO® vector (Thermo Fisher Scientific, MA, USA) following the manufacturer's instructions. The resulting plasmid, pTOPO::*tolR*, was transformed into chemically competent *Escherichia coli* DH5α cells for propagation and sequence-verified to confirm the correct insertion and orientation of the *tolR* gene.

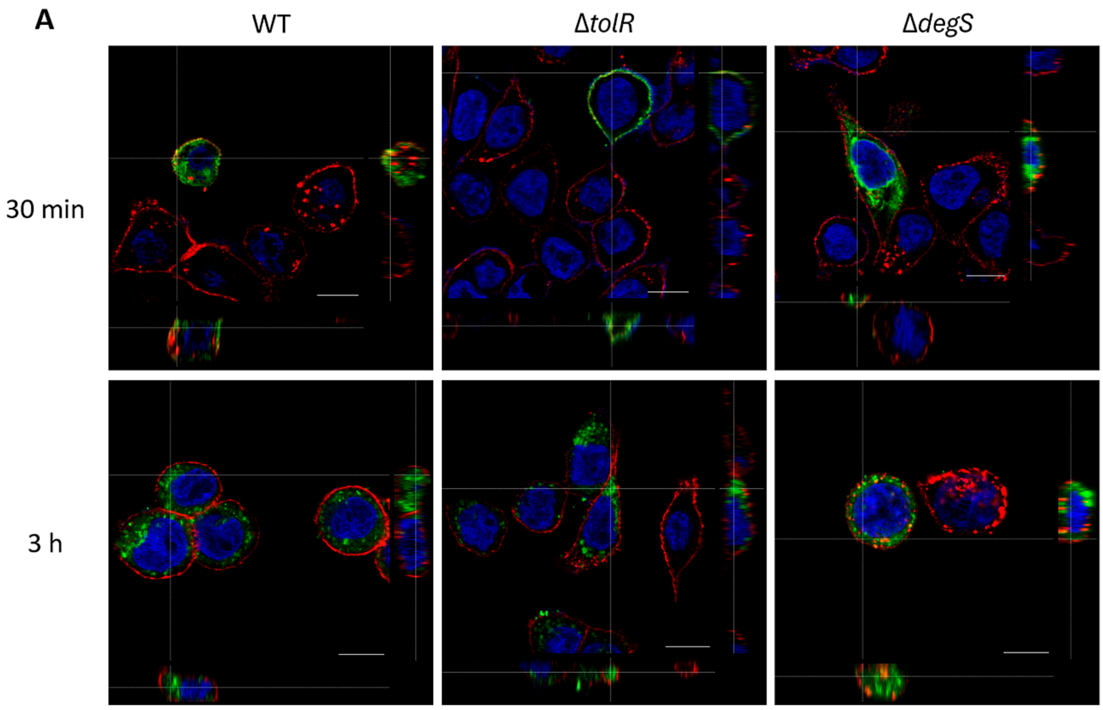

Figure S 4. Internalization of OMVs from *S.* Typhi WT, Δ*tolR*, and Δ*degS* strains by HT-29 cells. OMVs were fluorescently labeled with the lipophilic dye DiO (green) to track their localization and interactions with human epithelial HT-29 cells. Cells were counterstained with Hoechst to visualize nuclei (blue) and WGA to mark cell membranes (red). Confocal microscopy was performed to assess OMV uptake after 30 min and 3 h of incubation at 37 °C. Representative Z-stack projections are shown for each condition, illustrating the spatial relationships between OMVs and cellular structures. Scale bars: 10 µm. The results shown are representative of three independent experiments (n = 3).

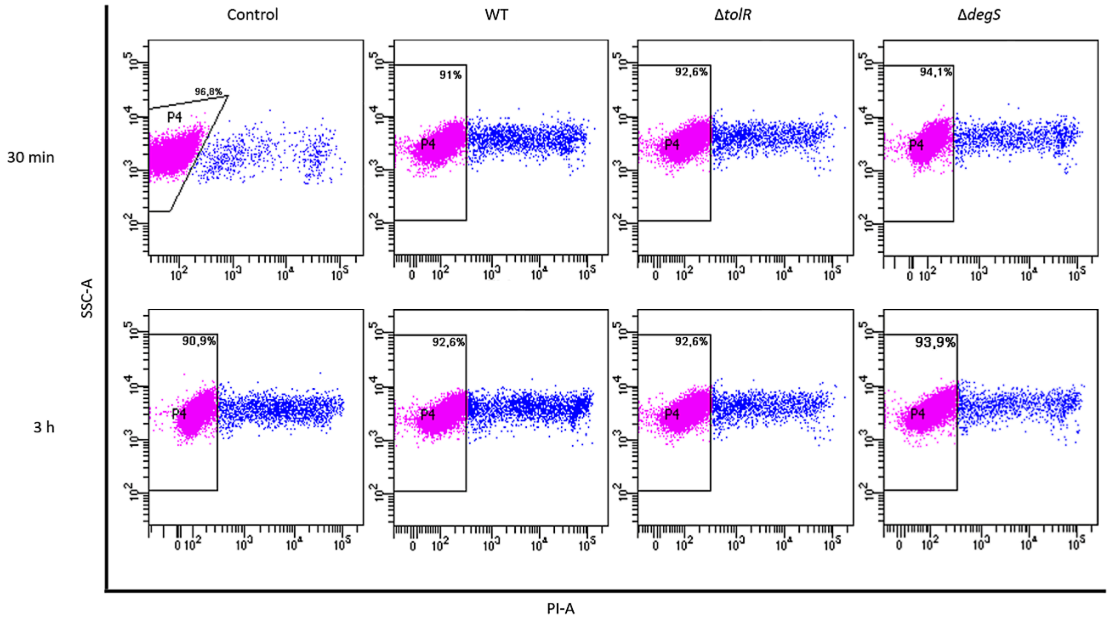

Figure S 5. Quantitative analysis of HT-29 cell viability after exposure to OMVs derived from *Salmonella* Typhi WT and mutant strains (Δ*tolR* and Δ*degS*). HT-29 cells were exposed to OMVs (100 µg/mL according to their protein content) for 30 min and 3 h. As a control, no OMVs were added. Cell viability was evaluated using propidium iodide (PI) staining, which selectively labels cells with compromised membrane integrity (PI-positive). Flow cytometry determined the proportion of viable (PI-negative, magenta) and non-viable (PI-positive, blue). The representative histogram displays data from three independent experiments (n = 3). All conditions tested showed consistently high viability rates (>90%), underscoring the minimal cytotoxicity of OMVs under the experimental conditions.

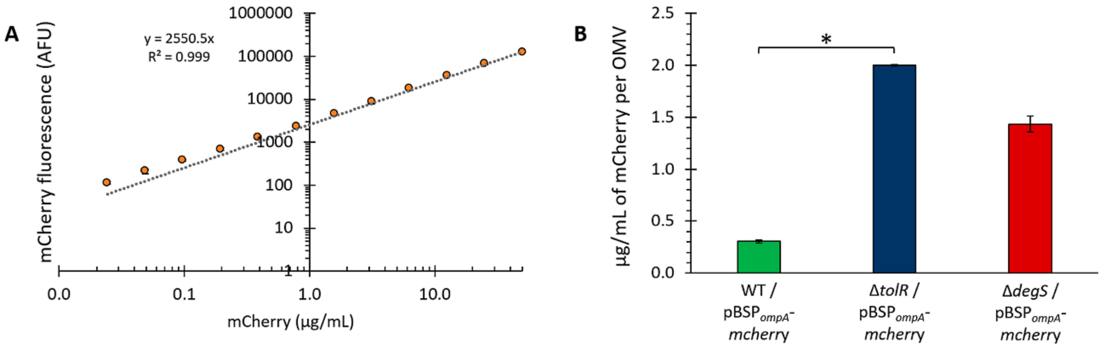

Figure S 6. Estimation of mCherry in OMVs for immunization studies in mice. **(A)** A calibration curve was constructed to quantify mCherry concentrations in OMV preparations based on fluorescence intensity measurements. Serial two-fold dilutions of purified mCherry were prepared. Using a Synergy H1 plate reader, fluorescence excitation and emission were measured at 587 nm and 630 nm, respectively. **(B)** Determination of mCherry in OMVs. Fluorescence intensities of OMV samples (excitation and emission at 587 nm and 630 nm, respectively) were interpolated onto the calibration curve to calculate mCherry concentrations in OMVs derived from *S.* Typhi WT, Δ*tolR*, and Δ*degS* strains.
